# Multifunctional Cellulose Nanofiber Films With Embedded Highly Oriented MXene Flakes

**DOI:** 10.1002/smll.74871

**Published:** 2026-08-04

**Authors:** Valeriia Poliukhova, Jacob Crossno, Kateryna Diedkova, Viktoriia Korniienko, Jisoo Jeon, Sankarshanaram S. Vempati, Jaejun Lee, Kirt A. Page, Hilmar Koerner, Maksym Pogorielov, Yury Gogotsi, Vladimir V. Tsukruk

**Affiliations:** ^1^ School of Materials Science and Engineering Georgia Institute of Technology Atlanta Georgia USA; ^2^ Materials and Manufacturing Directorate Air Force Research Laboratory WPAFB Dayton Ohio USA; ^3^ AV Inc. Dayton Ohio USA; ^4^ Biomedical Research Center Medical Institute Sumy State University Sumy Ukraine; ^5^ Institute of Atomic Physics and Spectroscopy University of Latvia Riga Latvia; ^6^ A. J. Drexel Nanomaterials Institute and Department of Materials Science and Engineering Drexel University Philadelphia Pennsylvania USA

**Keywords:** cellulose nanofiber films, organic dye adsorption, photothermal antibacterial properties, Ti_3_C_2_T_x_ MXene in‐plane alignment, ultrathin transparent nanocomposites

## Abstract

Ultrathin transparent photonic films that are simultaneously robust, cytocompatible, and actively antimicrobial remain rare. Here, the critical physical properties of freestanding micrometer‐thick films composed of layered cellulose nanofibers intercalated with Ti_3_C_2_T*_x_* MXene nanosheets (CNF‐MXene) are established. Correlated co‐alignment of MXene flakes with near‐perfect in‐plane order is achieved in the cellulose nanofiber matrix by vacuum‐assisted filtration. The Herman's orientation parameter of MXene nanosheets reaches up to 0.94, approaching the theoretical limit of 1.0 for perfect orientational order. Flow‐assisted alignment and nanofiber‐mediated confinement are proposed to suppress MXene restacking and lock the unique film architecture into a stable scaffold. This highly ordered structure yields a significant increase in mechanical strength and elastic modulus. Moreover, co‐alignment of individual flakes at ultralow volume fractions (below 1%) creates accessible, photothermally active surface sites within optically clear films. As a result, these ultrathin CNF‐MXene membranes combine near‐infrared‐activated photothermal antimicrobial behavior and molecular adsorption of organic dye as a proxy for accessibility, highlighting a distinctive multifunctional platform for active bio‐based films with strong potential for wound‐healing applications and long‐term functionality preservation.

## Graphical abstract

The critical physical properties of freestanding micrometer‐thick films composed of layered cellulose nanofibers intercalated with Ti_3_C_2_T*_x_* MXene nanosheets (CNF‐MXene) are investigated to find correlated co‐alignment of MXene flakes in the cellulose nanofiber matrix by vacuum‐assisted filtration.

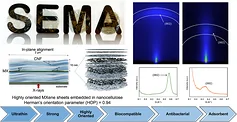

## 1 Introduction

The combined effects of material components enable unique mechanical, optical, electrical, sensing, self‐healing, and barrier properties that cannot be achieved in single‐phase films. This range of properties can be realized by customizing the composition and structure of combined organic or inorganic phases within polymer matrices. Boosting strength and toughness without sacrificing flexibility while relying on biocompatible and sustainable building blocks is an objective for advanced healthcare, wound dressings, and implantable devices. Integrating a combination of optical transparency, unique photonic properties, mechanical robustness, and active functional performance, e.g., antibacterial, sensing, catalytic, plasmonic, and barrier properties, in free‐standing ultrathin films, is a grand challenge. Ongoing research pushes composite thin films toward ever‐thinner, more defect‐free, highly aligned structures, including bio‐inspired nanocomposites, layered 2D materials, and multiphase stacks.

Recent advances highlight design principles borrowed from nature's bio‐inspired brick‐and‐mortar architectures, emphasizing the importance of interfacial interactions and adjusting nano‐ and microstructure, thereby promoting simultaneous stiffness, toughness, and added functionality at micrometer‐scale thicknesses [1]. Among the latest developments, bio‐composite nanomaterials emerged as a compelling platform where networks of cellulose nanocrystals and nanofibers can deliver photonic and emitting films with high transparency and good mechanical performance [2, 3, 4, 5]. Seamless matching with polymeric and inorganic materials was considered for diverse applications like optoelectronics, flexible devices, and water management [6, 7, 8]. In fact, bio‐derived and biocompatible cellulosic nanomaterials are known for their light weight combined with high tensile modulus and strength, achieved through drying and hydrogen bonding that cause the chains to cluster in a highly ordered structure [9, 10].

Reported mechanical parameters vary widely for cellulose materials with different processing conditions (e.g., filtration rate, drying, draw/bend alignment), underscoring that internal alignment, not just composition and surface groups, controls properties. 2D Ti_3_C_2_T*_x_* MXene nanosheets [11, 12], often highlighted for their exceptional electrical conductivity and photothermal properties, exhibit an effective elastic modulus of 330 GPa for monolayer flakes, making them an ideal building block for composite films, membranes, and coatings [13]. However, current freestanding MXene films are opaque and have a limited strain‐to‐failure and varied strength, which is dependent on the MXene quality and flake sizes [14, 15]. Integration of MXene into a polymer matrix requires strict control of internal nanoscale organization, porosity, orientation, and thickness to enhance mechanical properties without compromising MXene's unique features, such as photothermal activation [16]. Finally, blending MXene into polymer matrices with a high weight ratio is required for significant improvement [17, 18, 19, 20, 21].

In contrast to traditional polymer blending, integration within bio‐derived matrices, such as cellulose nanofibers (CNFs), can result in tailored composites due to the nature of semiflexible nanofibers [22, 23]. One challenge is the lack of flake‐alignment control, which often leads to a random or agglomerated network, failing to fully translate nanoscale properties into high‐performance macroscale behavior [24]. Another issue is compromised mechanical properties at high MXene content, resulting in brittle, stiff films that undermine the flexibility and strength that cellulose nanofibers would otherwise impart [25]. Most critically, however, MXene/polymer composites achieved substantial bacterial killing under near‐infrared (NIR) irradiation only with relatively high MXene fractions, as the photothermal effect scales with MXene concentration [26]. This dependence on high MXene content is problematic, as it exacerbates mechanical and processing issues and raises concerns about biocompatibility and cost.

In the recent study, 2D Ti_3_C_2_T*_x_* MXene flakes were integrated into layered cellulose nanofiber networks to demonstrate microscale photonic structural coloration of MXene flakes arising from local optical cavities [27]. The films were fabricated using vacuum‐assisted filtration (VAF), resulting in a uniform distribution of flakes within cellulose‐bundled nanofibers. The present work advances this platform by quantitatively establishing the unique co‐aligned architecture critically important for mechanical reinforcement and biointerface‐relevant transport and wound‐healing related functions. We establish the physical basis and effects of the anisotropic assembly of nanomaterials in the composites.

We suggest the unidirectional water flow during the fabrication process reorganizes both cellulose nanofiber matrix and 2D MXene flakes by solvent drainage that promotes high‐level alignment of the components parallel to the filter plane, where the CNF network actively suppresses out‐of‐plane orientation of the larger MXene flakes, suppressing restacking [28, 29]. The complete drying process fixes this structure through CNF‐CNF and CNF‐MXene interactions, consistent with the increased basal spacing and the near‐perfect Herman's orientation parameter (HOP) of ∼ 0.94 observed in the composites. This combination of flow‐assisted alignment and nanofiber‐mediated confinement provides a route to produce an inherent structural anisotropy and potentially novel physical properties relevant to biocompatibility, antibacterial activity, and molecular adsorption are also observed.

Clearly, a key technical gap in the field is finding a way to retain functional properties without compromising mechanical performance, transparency, and strong photothermal antibacterial activity, while maximizing efficiency and minimizing MXene content, which is a central idea of the current investigation. Specifically, the advance reported here is that near‐perfect in‐plane MXene order is quantified and shown to enable simultaneous transparency, mechanical reinforcement, and biointerface function at ultralow MXene loadings, distinguishing this work from high‐loading antibacterial MXene/polymer composites in prior research and literature data. The physical properties of these unique transparent CNF‐MXene composite films with ultralow MXene content (0.07% to 1.38% volume fraction) are examined, showing enhanced mechanical properties, including a significant increase in strength and a high elastic modulus of about 10 GPa.

## 2 Results and Discussion

**FIGURE 1:**
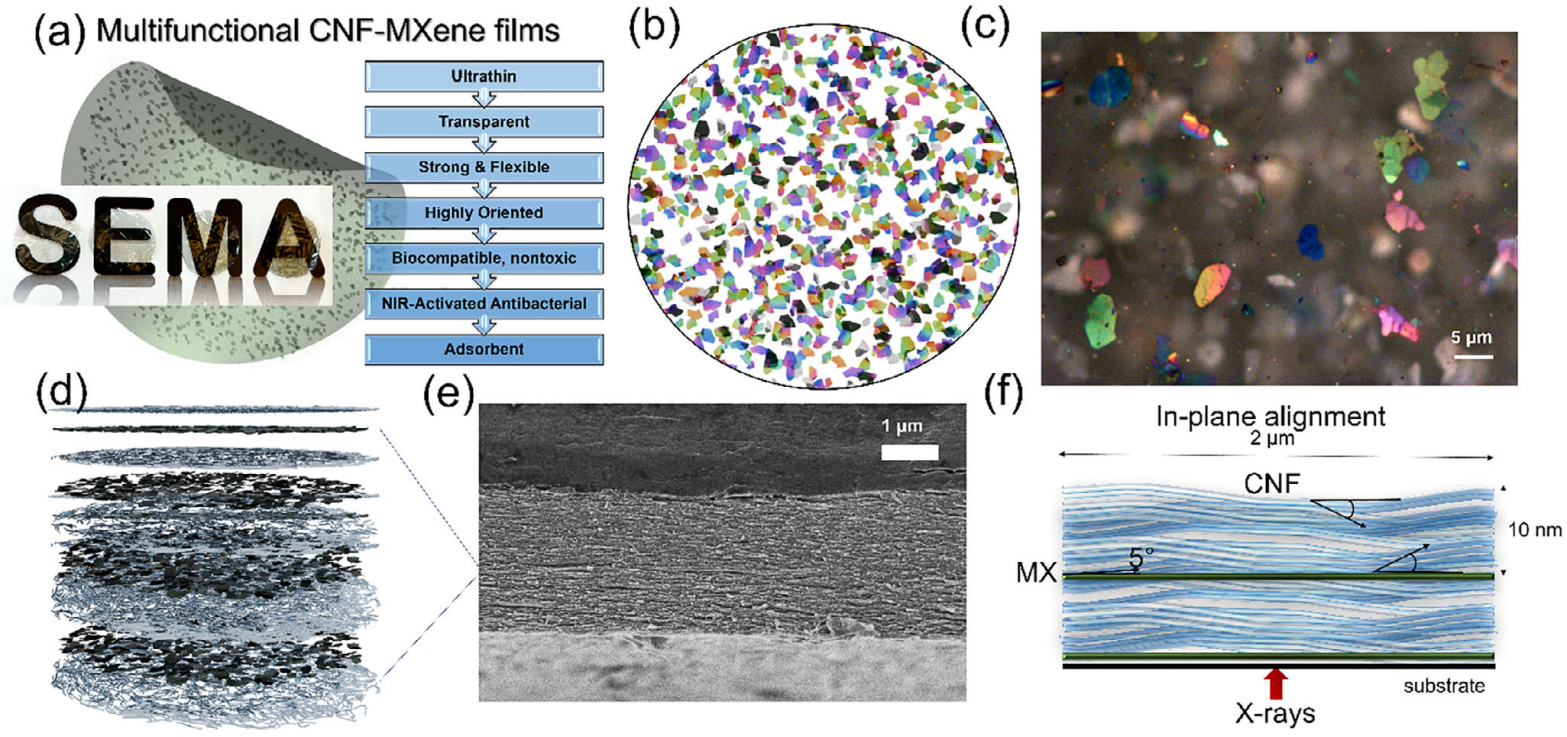
Schematic of a CNF‐MXene film with overlayed photographs showing MXene fractions from 0.07% to 0.28% (left to right) and their key attributes (a), schematic illustration of the multispectral structural colors of MXene flakes observed with an optical microscope of *CNF‐MX‐φ‐0.28%* (b), bright‐field unpolarized optical microscopy image of a CNF‐MXene film (c), schematic of the internal film structure with co‐aligned CNF fibers (light blue) interleaved with exfoliated MXene flakes (dark green) (d), scanning electron micrograph (SEM) cross‐section of *CNF‐MX‐φ‐0.28%* (e), and schematic of the interlocked orientational ordering of the material components illustrating the dominant in‐plane orientation of MXene (green) nanosheets within aligned nanofibers (blue) (f).

This work examines the functionality of photonic CNF‐MXene films to explore their physical properties beyond their photonic features (Figure 1) [27]. Building on the previously reported photonic behavior of these ultrathin CNF–MXene films, the present study focuses on quantitative structure–property–function relationships enabled by nanosheet alignment in nanocellulose matrix as a base for unique functional properties demonstrated here.

The amount of delaminated Ti_3_C_2_T*_x_* MXene in cellulose nanofiber network was varied by volume fractions (*φ*, from 0.07% to 1.38%). The CNF‐MXene films, with a thickness of 4 µm, were produced through vacuum‐assisted filtration (VAF), as detailed in a previous publication (Figure 1) [27]. As observed earlier, colorful MXene flakes were distributed within the cellulose matrix with color appearance controlled by the distance from the top surface (Figure 1a–c). The structural colors originating from local optical cavities are visible under the microscope because individual MXene sheets are well dispersed within the fiber network and lie horizontally [27]. Furthermore, a small volume fraction of MXene in the natural material composites, such as cellulose, is necessary to avoid excessive light absorption of stacked Ti_3_C_2_T*_x_* nanosheets and maintain transparency of the films, which is required in many functional applications (Figure 1a). Each CNF‐MXene film exhibits layered nanofiber‐bundle morphology and is associated with a stable colloidal suspension of the components, followed by homogeneous dispersion and hydrogen bonding between flakes and nanofibers upon drying, as shown in Figure 1d,e [27].

Initial mechanical testing suggested that the lamellae are in‐plane oriented, but further studies are needed to elucidate the effect of fine layered morphology in critical physical properties, as discussed here (Figure 1f). It is suggested that during VAF, the in‐plane alignment of the components emerges from coupled flow and confinement processes rather than from random deposition due to flow‐induced confinement at the membrane‐suspension interface. Through‐thickness solvent drainage imposes hydrodynamic drag on the high‐aspect‐ratio CNF suspension with MXene sheets, biasing them toward parallel configurations with respect to the filter plane under vacuum. As the CNF network progressively densifies, the available free space becomes anisotropic, restricting out‐of‐plane rotation of larger MXene flakes and thereby preventing restacking and suppressing stochastic tumbling as well as unfavorable out of plane orientation of nanosheets [28, 29]. Subsequent compression during drying further locks this geometry by hydrogen bonding and interfacial interactions of CNF‐CNF and CNF‐MXene.

### 2.1 Alignment and Orientation Morphology

**FIGURE 2:**
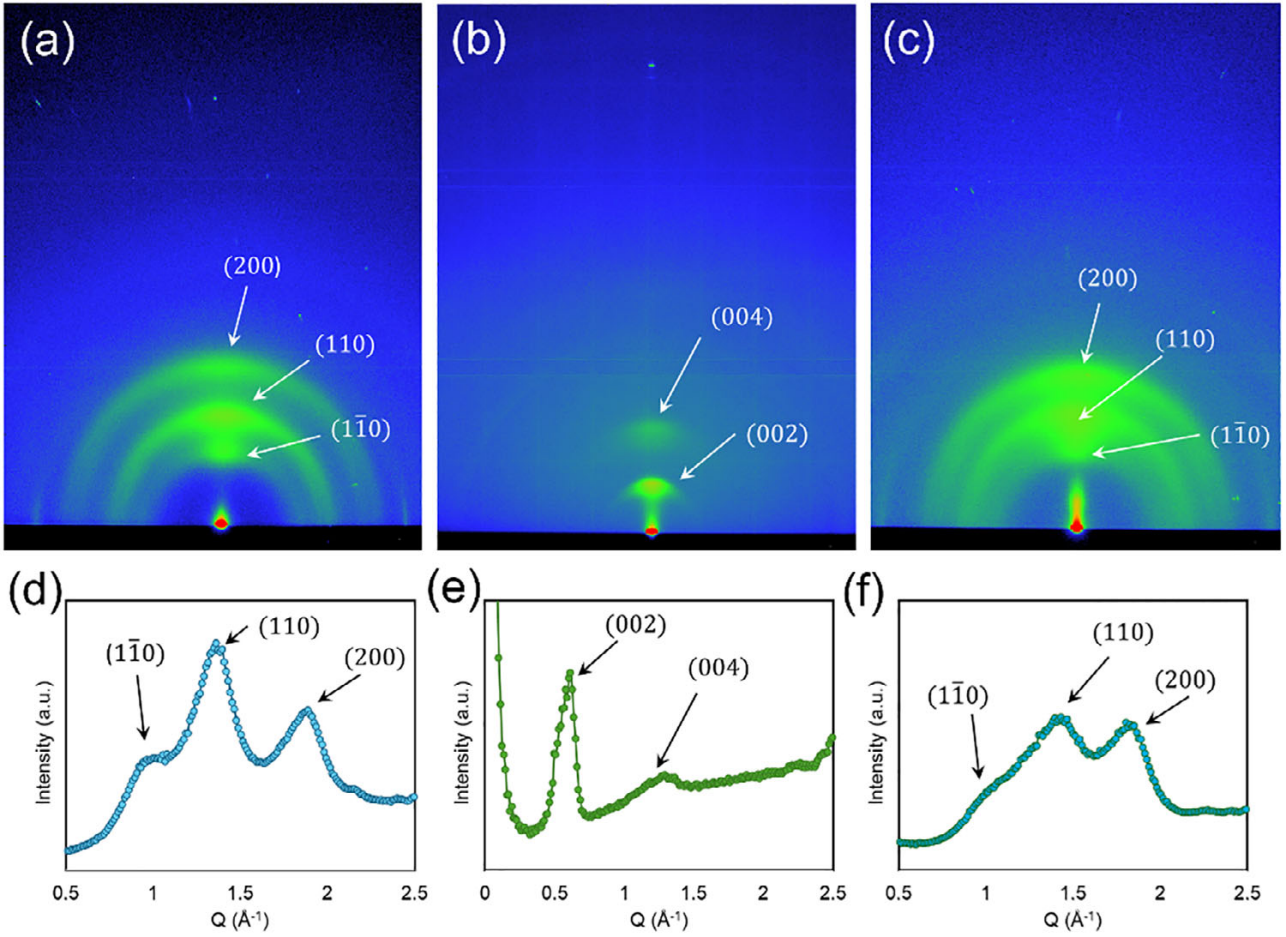
In‐plane GIWAXS 2D scattering images and radial vertical intensity plots (I vs. Q) comparison for the CNF (a, d), Ti_3_C_2_T_x_ MXene (b, e), and *CNF‐MX‐φ‐0.07%* (c, f).

To analyze the orientational ordering of nanofibers and nanosheets within films, grazing‐incidence wide‐angle X‐ray scattering (GIWAXS) and grazing‐incidence medium‐angle X‐ray scattering (GIMAXS) were employed (Figure 2). The GIWAXS results for CNF, MXene, and CNF‐MX‐φ‐0.07% are presented, with the lowest MXene content serving as a representative for all composite films (Figure 2). As evident from the X‐ray data, neat CNF films display the expected cellulose‐I reflections in the inner diffraction rings corresponding to (110) at *Q* = 1.35 Å^−^^1^ (*d*‐spacing of 4.65 Å), appearing the strongest and (200) at 1.87 Å^−^^1^ (*d*‐spacing of 3.36 Å), with narrow azimuthal lobes indicative of significant in‐plane alignment (Figure 2a,d) [30]. In‐plane GIWAXS 2D scattering images for all composite blends are presented in Figures S1 and S2.

Herman's orientation parameter (HOP) defines the macroscopic orientation of the film, where 1.0 is mathematically perfectly aligned, and 0.0 is characteristic of randomly oriented morphologies. The ‘near‐perfect’ orientation can be referred to parameters in the 0.90–0.95 range, and highly oriented in the 0.70–0.90 range, associated with a nearly saturated orientational order in highly drawn polymeric and layered systems, beyond which further processing yields only marginal increases [31, 32, 33, 34].

In the pristine MXene film, the GIWAXS pattern confirms a periodic lattice with a strong (002) basal reflection *Q* = 0.613 Å^−^^1^ and a secondary (004) reflection at *Q* = 1.29 Å^−^^1^ that corresponds to the expected basic *d‐*spacing of 14.2 Å, typical for non‐annealed MXene films fabricated from aqueous dispersions [35]. In the CNF films, the HOP for cellulose nanofibers, determined from the (110) reflection in the neat CNF film, is 0.47, suggesting that the high‐aspect ratio nanofibers are not entirely randomly oriented, as is typical for bulk CNF films. Instead, they are preferentially aligned parallel to the substrate after film formation through vacuum‐assisted suspension flow and subsequent drying. The HOP for the neat MXene film calculated from the (002) reflection azimuthal distribution is relatively high (0.81), indicating predominantly in‐plane alignment, but also noticeable restacking that prevents near‐perfect alignment in MXene films (Table S2).

In composite *CNF‐MX‐φ‐0.07%*, the GIWAXS pattern is essentially identical to that of neat CNF films with slightly decreased peak intensities, and no apparent MXene diffraction peak is observed, indicating possible intercalation of the nanofibers into MXene stacks (Figure 2c,f and Figure S1). Across the CNF‐MXene samples, the nanofiber orientation is statistically unchanged (Table S1), with an average HOP of 0.47‐0.53, as calculated from the (110) reflection. HOP is slightly down to 0.38 for the composite film with the highest MXene content (*CNF‐MX‐φ‐1.38%*), indicating a slight disruption of the preferred orientation, with more nanosheets embedded within cellulose nanofibers and possible localized MXene aggregation or restacking.

**FIGURE 3:**
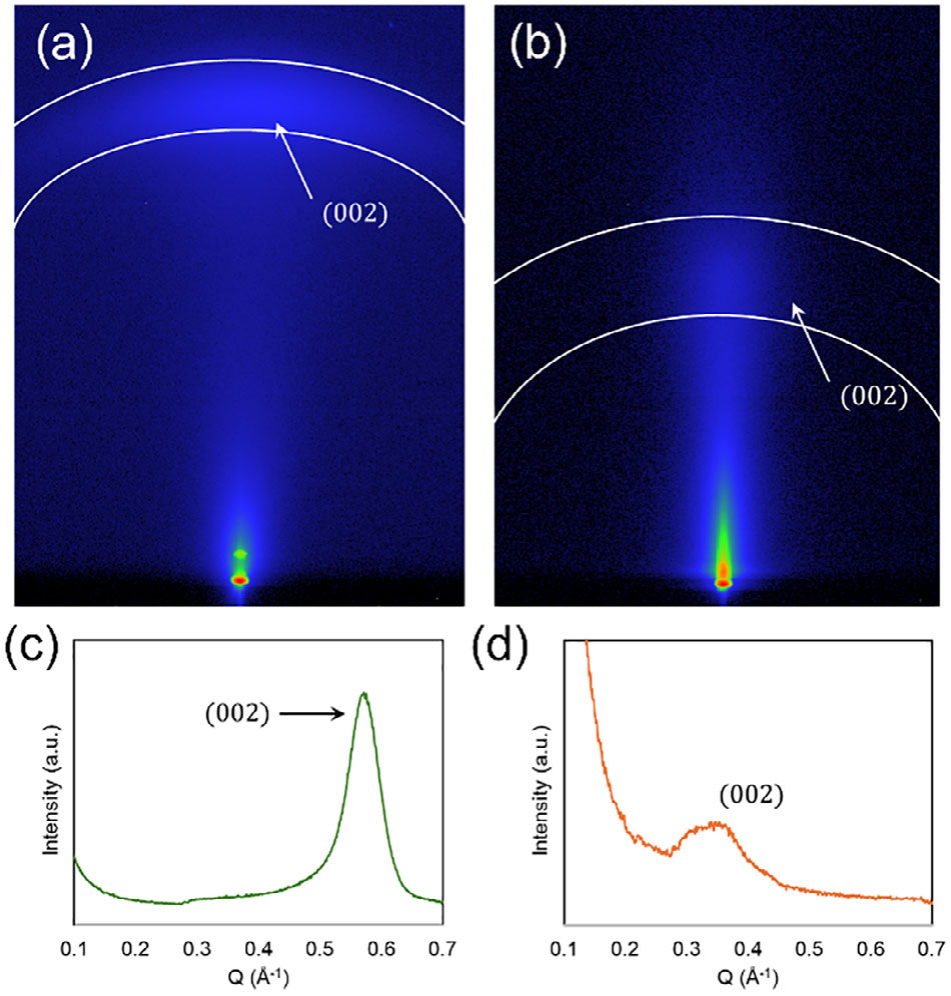
In‐plane GIMAXS 2D scattering images and radial vertical intensity plots (I vs. Q) comparison for the MXene film (a,c), and *CNF‐MX‐φ‐0.07%* composite film (b,d).

The effect of nanofiber intercalation on MXene flake distribution and planar orientation is most clearly observed in GIMAXS geometry, which provides higher resolution in reciprocal space (Figure 3a). Corresponding in‐plane GIMAXS 2D scattering images for all composite films are presented in Figure S3. GIMAX images reveal a shift of Bragg reflections toward higher d‐spacings, along with an increase in the orientation parameter. The neat MXene film shows (002) peak at *Q* = 0.60 Å^−^^1^ (d spacing 10.47 Å) (Figure 3c). In the *CNF‐MX‐φ‐0.07%*, the peak shifts to *Q* = 0.35 Å^−^^1^ (d spacing 17.95 Å), signifying major nanofiber‐mediated intercalation (Figure 3b,d). The HOP calculated from GIMAXS for the CNF‐MXene films with MXene φ of 0.07%, 0.28%, 0.42%, 0.69%, and 1.38% increases from 0.81 (neat MXene film) to 0.88, 0.92, 0.93, 0.94, and 0.90, respectively (Table S2). The observed increase in MXene basal spacing and the concurrent rise in HOP in the composites are consistent with confinement‐assisted alignment during VAF processing. Overall, GIMAXS demonstrates a consistent trend whereby all CNF‐MXene composites exhibit near‐perfect alignment of the flakes (0.88–0.94) within the cellulose nanofiber matrix. This suggests that the MXene sheets are predominantly oriented parallel to the film plane within the partially oriented CNF network, with azimuthal angle deviation typically maintained within 10°.

Furthermore, additional information can be deduced by comparing azimuthal intensity profiles (*I* vs. χ) of the MXene (002) reflection collected using GIMAXS, with the width of the azimuthal peak being inversely proportional to orientational ordering (Figure 3 and Figure S4). This comparison shows that the Ti_3_C_2_T*_x_* film has a relatively broad azimuthal distribution of the (002) peak (FWHM or Δχ = 12°). However, composite CNF‐MXene films display sharper azimuthal peaks (FWHM or Δχ < 10°). Among the composite films, *CNF‐MX‐φ‐0.69%* exhibits the narrowest peak (9.5°), indicating the highest nanosheet horizontal alignment, consistent with the direct HOP calculations discussed above.

### 2.2 Mechanical Performance

Here, the impact of in‐plane component alignment is evaluated, showing enhanced mechanical performance. Generally, a higher in‐plane orientation increases the load‐bearing fraction of cellulose fibers and MXene sheets, impacting Young's modulus and tensile strength, and the laminated architecture can raise toughness. Conversely, increasing MXene content can lead to restacking or interfacial defects, which decrease elongation at break. Mechanistically, the moderately aligned fibers in our CNF‐MXene films form a continuous hydrogen‐bonded network that facilitates axial load transfer, while MXene nanosheets serve as plate‐like structure reinforcements [27]. Efficient stress transfer across different components requires good interfacial adhesion and sufficient nanosheet separation to avoid shear‐lag saturation. Furthermore, the mechanical toughness can increase through the known mechanism of multiple crack deflection along nanofiber/nanosheet interfaces.

**FIGURE 4:**
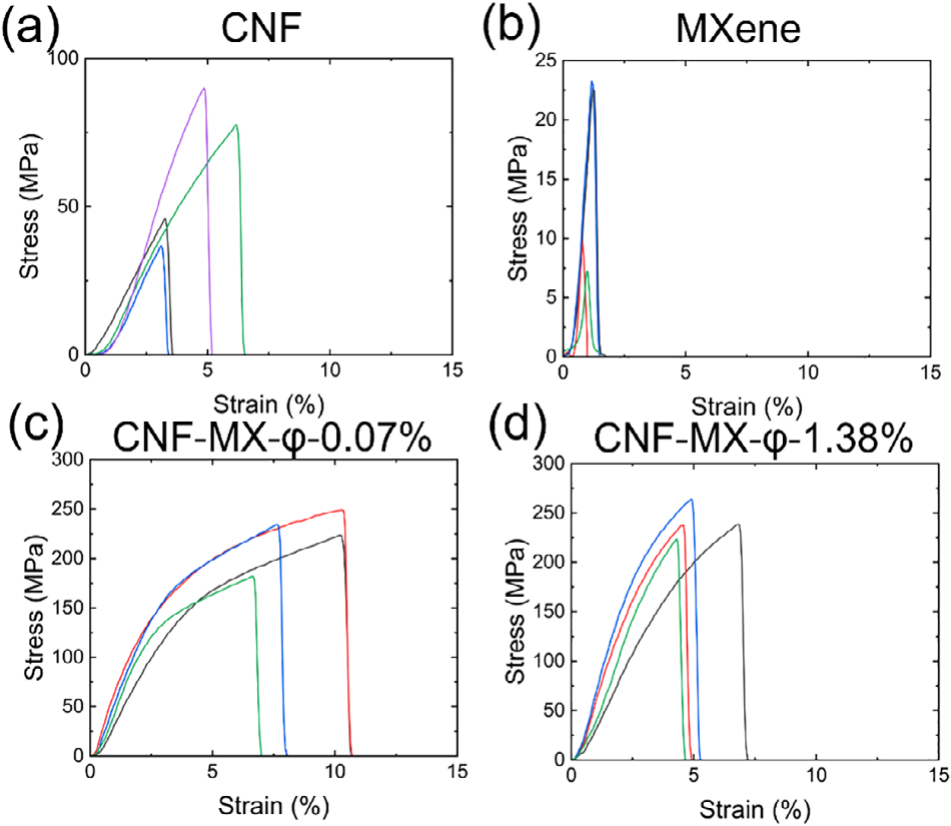
Measured stress–strain curves of CNF (a), MXene (b), *CNF‐MX‐φ‐0.07%* (c), and *CNF‐MX‐φ‐1.38%* (d) films.

To monitor changes in mechanical performance, we compare the stress–strain curves of CNF, MXene, *CNF‐MX‐φ‐0.07%*, and *CNF‐MX‐φ‐1.38%* films, both with an average thickness of 4 µm (Figure 4). Films with the lowest and highest MXene content among the composite samples were selected to highlight variations in stress–strain behavior, as the strength of the composite films remains within a similar range across different compositions. It was observed that in the neat CNF, the elastic deformation region exhibits a low slope followed by an extended strain‐hardening phase before failure, whereas MXene displays a significantly steeper initial slope with a shorter elastic regime and abrupt failure at small strain (Figure 4a,b).

The stress–strain curves for CNF, MXene, *CNF‐MX‐φ‐0.07%*, and *CNF‐MX‐φ‐1.38%* indicate that higher MXene content in the composite generally raises the initial stiffness and strength but progressively reduces elongation at break (Figure 4a–d). In *CNF‐MX‐φ‐0.07%*, the initial slope of stress–strain plots is noticeably higher than that of initial CNF films, indicating an efficient load transfer to a few well‐dispersed sheets. In the *CNF‐MX‐φ‐1.38%*, we observe the highest initial slope and peak stress among the four, but failure occurs at markedly lower strain with a sharper drop. Thus, the stiffness and strength of the composite films increase, while elongation at break decreases, a common trend in reinforced composite materials.

**FIGURE 5:**
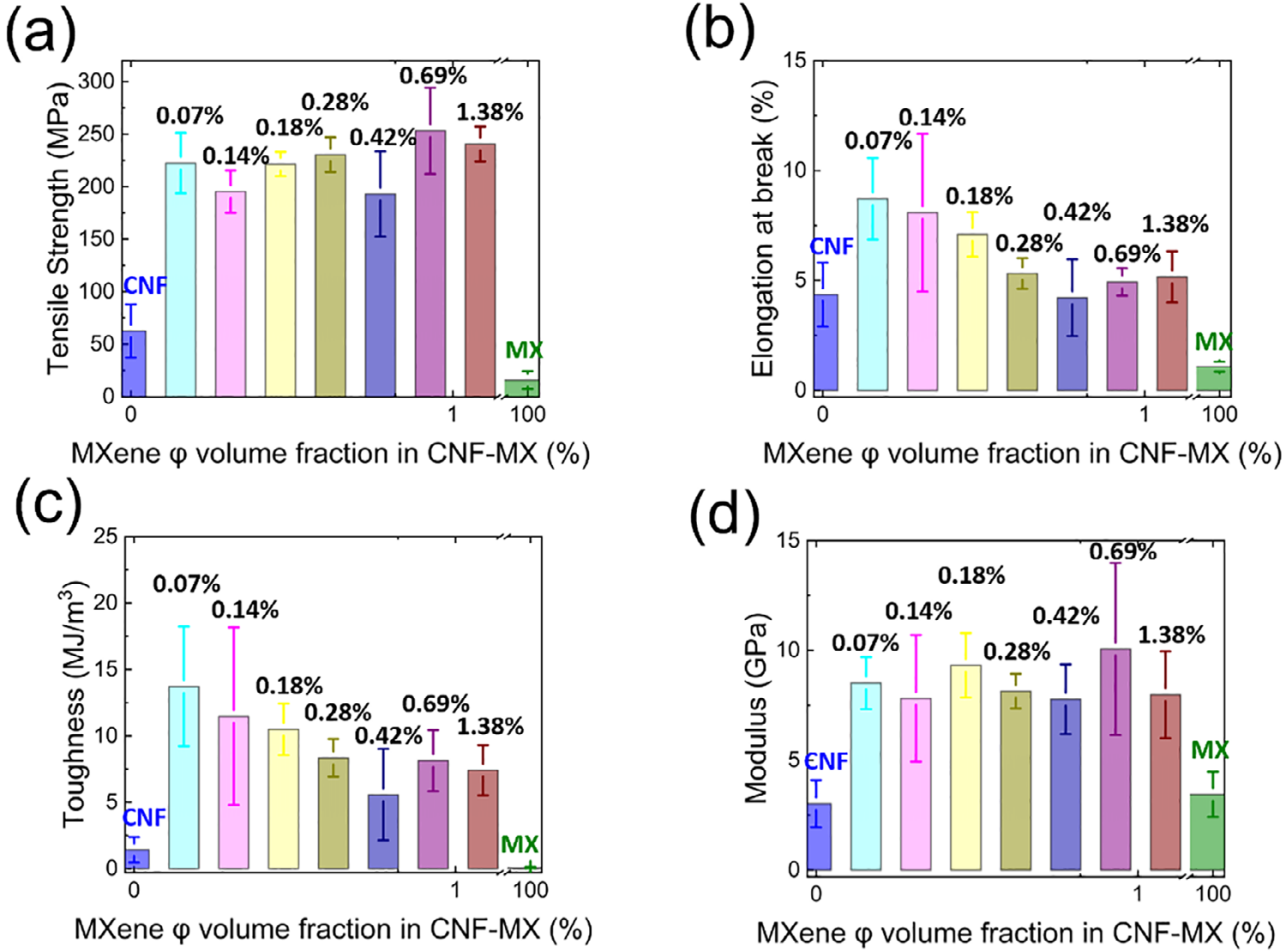
Variation of mechanical properties in the CNF‐MXene films of tensile strength (a), elongation at break (b), toughness (c), and modulus (d) vs. MXene φ volume fraction (from 0.07% to 1.38%) in cellulose network.

Tensile strength, elongation at break, and modulus show trends with MXene reinforcement in the cellulose nanofiber network (Figure 5). Mean values of all composite films on mechanical properties are summarized in Table S3, where for *CNF‐MX‐φ‐0.07%* has a modulus of 8.5 ± 1.2 GPa, tensile strength of 222 ± 28 MPa, elongation at break of 8.7 ± 1.9%, and toughness 13.7 ± 4.5 MJ/m^3^. Across all CNF‐MXene films, the average strength is ≈ 222 ± 24 MPa, which is ∼ 3.5 times higher than neat CNF and an order of magnitude (14‐fold) higher than neat MXene. These results support the GIWAXS/GIMAXS orientation analysis: in‐plane MXene alignment, with strong hydrogen bonding within the fibrillar matrix, enhances load transfer along the fiber direction. The composite films with the highest MXene nanosheet alignment order, HOP = 0.94, *CNF‐MX‐φ‐0.69%*, exhibited the highest modulus of 10 ± 3.9 GPa and tensile strength of 253 ± 41 MPa. The relatively larger standard deviation observed for modulus among samples is attributed to specimen variability typical of ultrathin freestanding films, where minor differences in local thickness, edge quality after cutting, and grip alignment can disproportionately affect the apparent modulus. We do not attribute this spread to macroscopic phase segregation, because the overall strength remains in the same high range across the composite series and the GIWAXS/GIMAXS measurements show a consistent orientation trend in CNF‐MXene composites. All values are reported as mean ± standard deviation from five independent specimens per composition (*n* = 5), with variable specimen thickness as measured individually (within 3–4 µm). The larger scatter observed for modulus values in selected compositions is attributed to the sensitivity of ultrathin freestanding films to small variations in local thickness, edge quality after film cutting, and grip alignment, which disproportionately affects the initial elastic slope in very thin films, in contrast to traditional bulky specimens. Considering these experimental challenges scattering of values measured of around 30% can be considered as acceptable reproducibility, and it does not affect trends and conclusions.

This mechanical performance is comparable to that reported earlier for thicker CNF films with high MXene content. Indeed, other CNF‐MXene composite materials, such as MXene/CNF with porous carbon (PC) 20 µm film, exhibited a tensile strength of 38.6 MPa [36]; while paper of MXene with 60% CNF of 19 µm had tensile strength of 271.5 MPa and Young's Modulus of 18.5 GPa [25]. In another work, MXene/CNF film with silver nanowires (Ag NWs) had a tensile strength of 137 MPa for a 20 µm film [37]. The reported value differences are due to the composite's fabrication process, including its type, composition, ratio, and the degree of co‐alignment between MXene nanosheets and polymer components.

The trends presented in Figure 5 are consistent with alignment‐enabled load transfer within the layered brick‐and‐mortar‐like architecture of the composite. In the very low φ (0.07%–0.69%) range, modulus and tensile strength increase alongside the rise in MXene HOP (from 0.88 to 0.94), suggesting more effective stress sharing across hydrogen‐bonded CNF–MXene interfaces associated with improved nanosheets co‐aligning (Figure 5a,d). Elongation at break decreases with increasing MXene content, constraining the cellulose network by reducing rotation and sliding; thus, earlier cracks can initiate at restacked‐sheet contacts, leading to failure at lower strain (Figure 5b). Toughness is higher at the lowest MXene φ (0.07%–0.18%), where larger nanofiber content enhances load transfer and strength; as MXene content increases further, more brittle behavior is observed. (Figure 5c). At low MXene fractions, the CNF network remains continuous and deformable, permitting limited fibril sliding, where MXene nanosheets act as stiff, load‐bearing reinforcements that are sufficiently separated, thereby avoiding stresses from direct flake‐flake contacts, interfacial debonding, and crack deflection and propagation, and dissipating energy prior to final failure. As the MXene fraction increases, local restacking restricts matrix mobility and provides earlier crack‐initiation sites. This explains why elongation at break decreases with increasing MXene loading, while toughness is highest at the lowest MXene fractions (0.07%–0.18%), and films become more brittle at higher loading.

### 2.3 Antibacterial Properties and Biocompatibility

Ti_3_C_2_T*_x_* previously displayed excellent photothermal conversion efficiency under NIR irradiation, reaching 100% internal light‐to‐heat conversion [38, 39, 40], producing hyperthermia that promotes the ablation of bacterial structures [23]. MXene absorbs light across the visible spectrum, making free‐standing films opaque (deep purple) and scattering a significant fraction of the incoming radiation, undermining efficient photothermal bacteria inactivation. Moreover, MXenes alone are prone to high degradation rates in low concentrations and aqueous or moist environments, the cytotoxicity of which has been associated with the oxidation process [41, 42, 43]. Previous reports demonstrated composite multifunctionality by adding a third component, such as Ag nanowires [37] or conducting polymers [44]. For example, 100% kill of *E. coli* and *S. aureus* under 808 nm NIR (1.5 W/cm^2^) in <60 s was achieved in a CNF/MXene film with the addition of polyaniline (PANI); however, mechanical tests have not been performed. The nacre‐like CNF/MXene@Ag film exhibited a tensile strength of 71.4 MPa and excellent photothermal properties with a surface temperature of 48°C after 210 s of 55 W light irradiation [45]. Thus, combining MXene with polymers is a common strategy to boost mechanical properties, improve biocompatibility, mitigate oxidation effects, and stabilize functional performance, while using a fraction of the material and lowering production and large‐scale manufacturing costs.

**FIGURE 6:**
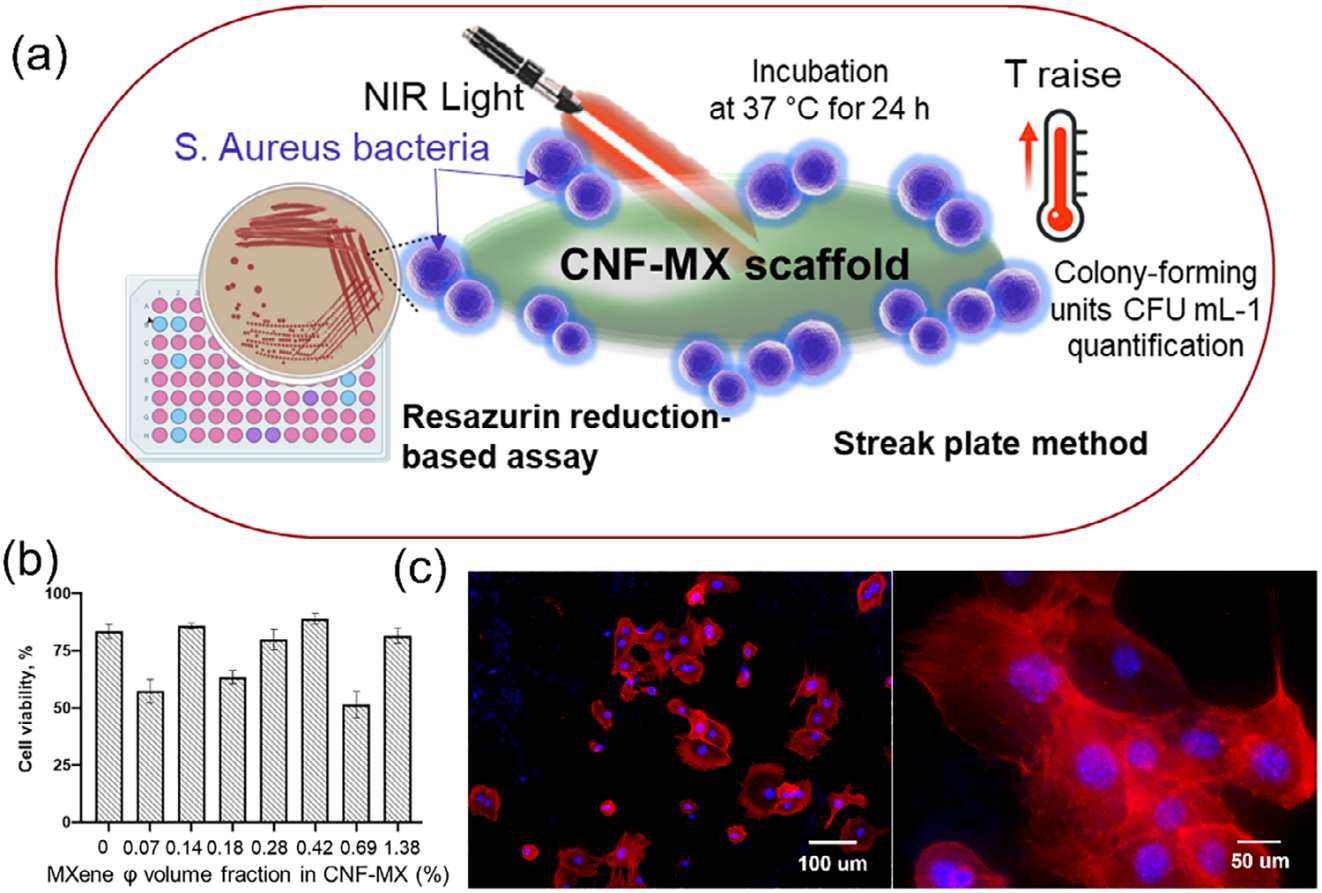
Schematic illustration depicting the resazurin reduction assay and photothermal ablation process of planktonic bacteria using CNF‐MXene scaffold as support (a), resazurin reduction assay data on the cytotoxicity of mouse embryonic fibroblasts during the 3‐day experiment on CNF and *CNF‐MX‐φ‐0.07‐1.38%* films (b), fluorescent images of nuclei (blue) and cytoskeleton staining (red) on the third day of cultures on the *CNF‐MX‐φ‐1.38%* (c).

The cytocompatibility and photothermally triggered antibacterial properties of CNF‐MXene used as a scaffold in this study are summarized in Figure 6a. A resazurin reduction assay was employed to evaluate the cytocompatibility of CNF‐MXene films (Figure 6b,c). Mouse embryonic fibroblasts adhered and spread on all surfaces of neat CNF and CNF‐MXene films with ultralow MXene loadings, superior to prior published research with high MXene content. Metabolic activity after 72 h typically fell in the 70%–85% range, and some wells showed values as low as ∼ 50% or as high as ∼ 85% (Figure 6b). These fluctuations did not track with increasing MXene content; the compositions differ only marginally and are below the sensitivity at which a monotonic viability trend would be expected. Instead, the scatter is consistent with well‐to‐well variability (e.g., slight differences in seeding/contact area) and with a noted artifact: partial disruption of the ultrathin CNF support during 4% formaldehyde fixation, which can detach adherent cells and depress the readout. When averaged across samples, all CNF‐MXene films exhibited ≥75% viability, indicating no acute cytotoxicity over 72 h. Fluorescence microscopy validated these findings, showing intact fibroblast morphology across all surfaces studied here. For demonstration, a representative image of the surface of the *CNF‐MX‐φ‐1.38%* scaffold is shown in Figure 6c, and all images are shown in Figure S5.

**FIGURE 7:**
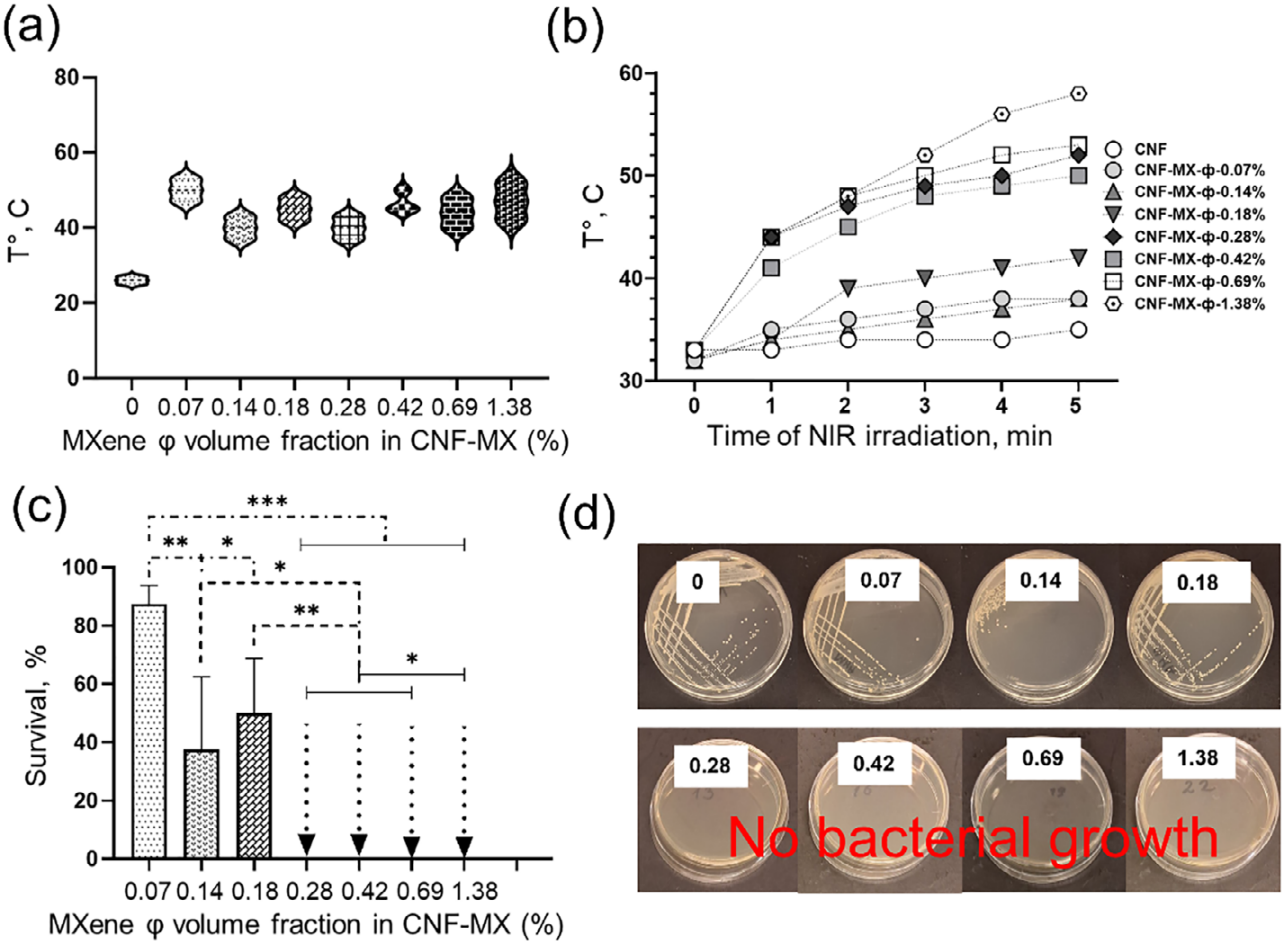
Average temperature (T) recorded after 5 min of 808 nm at 2 watts/10 Hz laser exposure (a), and T vs. time of NIR irradiation (b) using CNF and *CNF‐MX‐φ‐0.07‐1.38%* films, relative viability of planktonic bacteria (c), and digital images of *S. aureus* colonies (d) after co‐cultivation followed by laser exposure. Error bars represent standard deviation: ^*^ *p* < 0.05, ^**^ *p* < 0.01, ^***^ *p* < 0.001.

As the next step, to probe photothermal functionality relevant to infection control, films were irradiated for 5 min with an 808 nm laser, exhibiting a reproducible temperature rise from 23°C–26°C in the CNF to 42°C–58°C in the CNF‐MXene films (Figure 7a,b and Figure S6), confirming efficient light‐to‐heat conversion. Critical temperature growth was observed in samples above >0.28% *φ* MXene, indicating a minimum critical amount of MXene needed in an ultrathin cellulose scaffold, reflecting a balance between volumetric heat generation in the MXene and heat losses to the surrounding medium. Finally, at ultralow loadings (*φ* = 0.07%–0.18%), MXene flakes are well dispersed, but the source density is insufficient to exceed heat losses from the film interface; the measured temperature rise remains limited by heat losses to the surrounding medium, and any microscale temperature gradients near individual absorbing flakes are not resolved by the IR measurement.

Furthermore, above 0.28% φ, flake‐to‐flake spacing within the cellulose layers decreases, thereby increasing the effective optical path through the MXene and delivering more spatially uniform photothermally‐initiated local heating.

Against surface‐attached *S. aureus* bacteria, MXene alone (in the dark conditions) produced little to no inhibition. However, concurrent NIR exposure yielded a loading‐dependent reduction in recovered CFU relative to the CNF (Figure 7c,d), with the strongest effect observed for *CNF‐MX‐φ*‐0.28–1.8%. This result indicates that the minimum amount of MXene in the thin CNF scaffold (4 µm) should be ≥0.1 mg to enable efficient heat dissipation into the medium protected by the extracellular cellulose nanofiber matrix. The robust antibacterial response presented here underscores the high utilization efficiency achieved by uniform dispersion and in‐plane alignment of MXene flakes. Simultaneously, the co‐aligned architecture enhances in‐plane thermal spreading, promoting more homogeneous surface temperatures and more effective heat transfer into the biofilm/liquid boundary layer. While the bulk solution reached only 42°C–58°C, which is below the threshold typically associated with rapid biofilm ablation. The measured temperature represents a macroscopic average and may underestimate transient interfacial heating at the film–biofilm boundary that is not readily detectable. This indicates that the local MXene temperatures within the CNF‐MXene membrane and on its surface should exceed those of the surrounding medium [46]. Thus, CNF‐MXene scaffolds are cytocompatible, support fibroblast adhesion, and can provide sufficient microscale heating upon NIR light irradiation to damage the bacterial cultures despite macroscopic temperature limits.

### 2.4 Wound Healing Application

The envisioned wound‐healing application of the CNF‐MXene membrane entails direct exposure to a moist biological environment, raising concerns about MXene stability and potential oxidation in aqueous and oxidizing conditions, especially at low MXene concentrations. This can be controlled by limiting light and oxygen exposure, and it is the slowest in solid media, including polymer matrices [47]. In this context, the cellulose matrix of our CNF‐MXene thin films plays a dual stabilizing role, acting as a polymer host that embeds and spatially separates MXene flakes, limiting exposure to oxidizing species, and as a partially protective barrier that restricts direct access to the MXene surface during storage and handling. The UV–Vis spectrum of the representative scaffold with the minimum amount of MXene required for NIR activation, *CNF‐MX‐φ‐0.28%*, is used as a sensitive indicator of potential oxidation‐related spectral changes (e.g., UV–Vis peak shifts), which remain essentially unchanged after 1 year of storage under ambient dark conditions (Figure S7).

From an application standpoint, the CNF‐MXene membrane is designed for single‐use photothermal activation, where it is irradiated once per treatment session and serves as a passive protective barrier between the wound and the external environment until routine dressing replacement. Therefore, repeated NIR cycling of the same membrane is not required for the intended clinical use scenario. Importantly, our experimental protocol already includes prolonged exposure to an aqueous environment prior to NIR activation. Specifically, membranes were incubated in Mueller‐Hinton broth for 18 h at 37°C before 808 nm laser irradiation, yet they still exhibited a reproducible temperature rise and strong antibacterial efficacy, as discussed earlier (Figure 7). We performed an additional long‐term experiment using *CNF‐MX‐φ‐0.28%* sample stored for one year in the dark (sealed, room temperature), that was subsequently incubated in Mueller–Hinton broth for 18 h at 37°C and then irradiated under identical 808 nm conditions; the photothermal response and temperature elevation remained essentially unchanged compared with the freshly prepared film (Figure S8), indicating no measurable loss of photothermal performance effectiveness.

### 2.5 Molecular Adsorption for Surface Accessibility

Molecular adsorption of an organic dye was used here as a model assay to quantify accessible surface sites and transport through the aligned lamellar architecture. Rhodamine 6G (Rh 6G) serves as a convenient cationic chromophore with well‐defined UV–Vis and fluorescence signatures, enabling direct comparison of adsorption kinetics between CNF‐only and CNF–MXene membranes. The results, therefore, provide a quantitative evaluation of how nanosheet alignment and intra‐membrane organization expose MXene surfaces within the transparent CNF scaffold.

Most published ‘high‐capacity’ MXene composites rely on millimeter‐thick hydrogels, aerogels, or foams containing 5–10 wt.% of MXene. For example, cellulose/MXene/PVA composite reports ∼ 240 mg g^−1^ Methylene Blue adsorption capacity, due to its porous structure, which carries a large mass fraction of the MXene phase [48]. The surface of Ti_3_C_2_T_x_ has negative –O/–OH/–F terminations and mainly adsorbs cationic dyes through electrostatic attraction and *π–π* interactions, as determined by multiple studies [37, 44, 48]. TEMPO‐oxidized CNFs can act as hydrophilic adsorbents where hydrogen bonding and electrostatics dominate Rh 6G dye uptake [25]. Thus, investigating adsorption can help understand the surface sites accessibility and nanoscale transport properties within the lamellar films.

**FIGURE 8:**
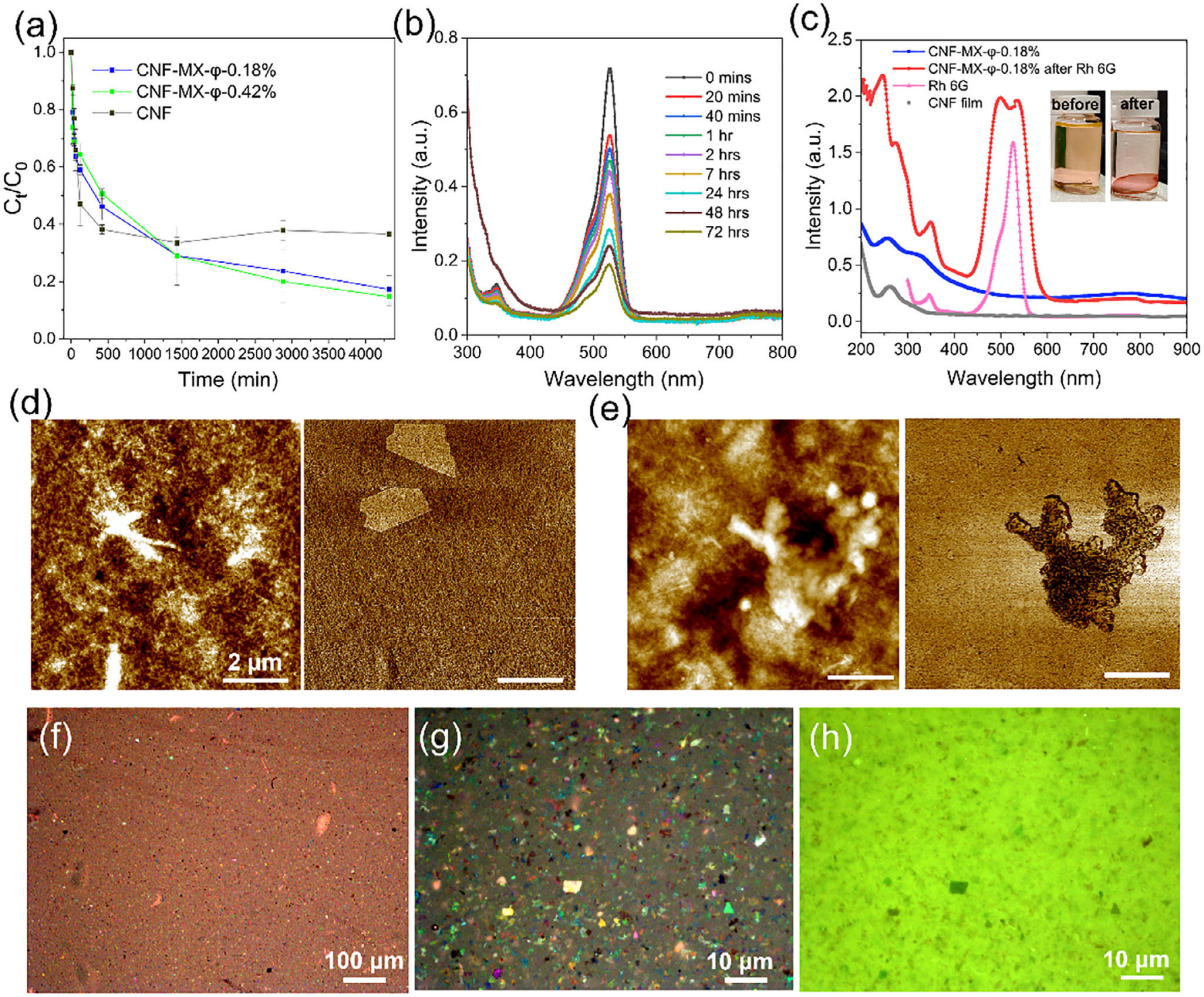
Dye adsorption kinetics, spectroscopy, and surface imaging: normalized dye concentration C_t_/C_0_ vs. time for CNF, *CNF‐MX‐φ‐0.18%* and *CNF‐MX‐φ‐0.42%* films (a), time‐resolved UV–Vis spectra of dye solution showing Rh 6G absorption intensity decay with *CNF‐MX‐φ‐0.18%* (b), solid‐state UV–Vis spectra before and after adsorption of Rh 6G on the *CNF‐MX‐φ‐0.18%*, solid spectra of the CNF film and Rh 6G solution at time 0 with photographs at 0 and 72 h (c); AFM topography and phase images of *CNF‐MX‐φ‐0.42%* before (d) and after (e) dye capture, with scale bars (d) 2 µm, Z‐scale 20 nm, phase 30°, and (e) Z‐scale 85 nm, phase 50°; optical microscopy of *CNF‐MX‐φ‐0.42%* after adsorption in low (f) and high resolution (g), and fluorescence image of (g) with 500 nm filter (h).

Molecular capture was evaluated as a proof of concept using Rh 6G of the *CNF‐MX‐φ‐0.18%* and *CNF‐MX‐φ‐0.42%* films (Figure 8). These results were compared to a control CNF‐only film. Parallel monitoring of surface property changes before and after Rh 6G adsorption was performed. The *CNF‐MX‐φ‐0.42%* was selected as the film with the highest MXene content for evaluation, as, after the Rh 6G adsorption tests, films become less transparent, making it difficult to measure. The composite films show a similar trend in removing Rh 6G from solution over a 72‐h period in the dark, but they sustain uptake beyond the rapid, early‐stage uptake seen for CNF films (Figure 8a). The fast initial drop in C_t_/C_0_ at short times can be attributed to the adsorption of Rh 6G on CNF hydroxyl and carboxylate sites. In contrast, the CNF‐MXene films reflect higher adsorption capacity even with negligible addition of MXene per film volume fraction (0.18% or 0.42%), which is due to additional binding on the exposed MXene surfaces intercalated within CNF fiber networks. This trend, therefore, combines dual‐site adsorption, with CNF carrying the initial uptake and MXene sustaining capacity after all available CNF sites have been occupied.

The Rh 6G removal efficiency increases substantially with MXene content: for the CNF film, it is 65%, rising to 77% for *CNF‐MX‐φ‐0.18%* and 89% for *CNF‐MX‐φ‐0.42%*. The gravimetric capacities (per gram of composite) for the adsorption of Rh 6G are: *CNF‐MX‐φ‐0.18%* = 24.2 mg g^−^^1^, *CNF‐MX‐φ‐0.42%* = 31.8 mg g^−^^1^, and CNF = 21.6 mg g^−^^1^. The volume fractions were converted to mass fractions using the bulk densities of MXene and CNF, and the composite capacities were normalized to the MXene mass fraction. This yields 4.82 × 10^3^ mg g^−^^1^ Rh 6G dye adsorption per MXene mass in the *CNF‐MX‐φ‐0.18%*, meaning that each gram of MXene embedded in the aligned CNF scaffold is removing grams of dye, reflecting the importance of individual MXene flakes with high‐site exposure due to homogeneous distribution within the nanocellulose network. Because CNFs are wettable and MXene nanosheets are aligned within, the Rh 6G dye can access MXene surfaces much faster with the µm diffusion paths, compared to stacked MXene nanosheets that block most adsorption sites.

The Rh 6G monomer band at 526 nm and its blue‐shifted shoulder at 495 nm, due to H‐type aggregation, decrease monotonically over 72 h, directly mirroring dye removal from solution, as evidenced by the recorded intensity changes (Figure 8b).

After exposure to Rh 6G, the *CNF‐MX‐φ‐0.18%* film develops a broad absorption feature in the 450–600 nm region (red curve), resembling the absorption spectra of Rh 6G dye (pink curve) while preserving the peaks attributed to CNF and MXene in the *CNF‐MX‐φ‐0.18%* film before adsorption (blue curve) (Figure 8c). The components remain intact after 72 h exposure to Rh 6G, preserving the CNF‐MXene signature. Rh 6G is immobilized on the surface, with band shape indicating a mix of surface‐bound monomers and small aggregates.

AFM topography and phase images of the *CNF‐MX‐φ‐0.18%* film surface before and after adsorption of the dye reveal the appearance of nanoscale deposits of crystallized Rh 6G dye; while the phase contrast emphasizes the distinct mechanical and viscoelastic properties of MXene relative to the CNF matrix (Figure 8d,e). Bright‐field optical micrographs of the adsorbed Rh 6G on the *CNF‐MX‐φ‐0.18%* film surface show a homogeneous pink hue over the film's surface and evenly distributed MXene flakes within the layers, which preserved its multispectral structural colors appearance [27] (Figure 8f,g).

At the same time, the corresponding green‐channel fluorescence confirms spatially uniform dye capture across the film (Figure 8h). A no‐dye control acquired under identical imaging conditions showed no detectable fluorescence, confirming that the observed signal in Figure 8h does not arise from background emission of the CNF matrix. The kinetic trend, spectroscopy data, and optical imaging provide a coherent picture of the added physical functionality, emphasizing effectiveness at ultralow MXene loadings in the CNF‐MXene films explored in this study.

## 3 Conclusions

In conclusion, we demonstrated the distinct high‐level co‐alignment of nanocellulose and flake components in CNF‐MXene films, reaching an orientation parameter of up to 0.94 for MXene nanosheets and expanded basal spacing indicative of partial cellulose intercalation while reducing restacking and maximizing the exposure of adsorption and photothermal sites. Notably, this alignment occurs at ultralow MXene concentrations, enabling the preservation of unique optical clarity for MXene‐based composites with the appearance of additional functionalities.

Indeed, this film morphology directly influences the functionality of CNF‐MXene composites in practical applications. Mechanical reinforcement is indicated by increased average modulus (8.5 ± 2 GPa), tensile strength (222 ± 20 MPa), ∼ 7% elongation at break, and ∼ 8 MJ/m^3^ toughness. Additionally, properties such as cytocompatibility (≥75% viability at 72 h), on‐demand photothermal antibacterial activity (>99% reduction of *S. aureus*), and organic dye adsorption (up to 89% removal efficiency) are all observed to amplify for these multifunctional membranes.

Remarkably, this multifunctionality is achieved with extremely low MXene concentrations, enabling efficient deployment of the active phase with exposed surface sites and heat dissipation facilitated by internal alignment. It is proposed that the co‐aligned morphology promotes in‐plane thermal distribution, with homogeneously distributed MXene flakes (∼0.1 mg within cellulose multilayers) rapidly heating under NIR laser illumination to eradicate bacteria, thereby mitigating the typical trade‐off between photothermal performance and mechanical flexibility or biocompatibility in thin films. This performance is comparable to or exceeds that of earlier developed composites with higher MXene contents, representing a notable advancement in the field. The use of minimal MXene loading addresses practical considerations such as cost and material availability, thus supporting scalable manufacturing.

High dispersion and alignment of nanosheets within the cellulose nanofiber scaffold directly impact film properties, including biocompatibility, antibacterial activity, mechanical integrity, and active‐material efficiency. The findings contribute to establishing structure‐property‐function relationships that facilitate the development of transparent, robust, photothermally active antibacterial scaffolds and potential molecular adsorption platforms. Beyond wound care, the alignment‐guided strategy can be applicable to critical flexible biointerfaces such as on‐skin electronics, bioelectronic devices, antifouling membranes, and cytocompatible photothermal coatings.

## 4 Characterization Techniques and Methods

### 4.1 Materials Synthesis

Details regarding CNFs and Ti_3_C_2_T_x_ MXene, including their synthesis, parameters of individual fibers and flakes, as well as CNF‐MXene film fabrication and composition, were provided in a previous work and supporting materials [27].

### 4.2 GIWAXS/GIMAXS Measurements and Analysis

Grazing‐incidence X‐ray scattering measurements were performed using a Xenocs Xeuss 3.0 SAXS/WAXS system equipped with a Pilatus3 R 300K detector. Measurements were collected using an incident x‐ray wavelength of 0.154 nm at sample‐to‐detector distances of 0.06, 0.6, and 1.6 m for each sample. All samples were mounted on clean silicon wafers and measured at an incident angle of 0.1°. For the GIWAXS data, the scattering is shown as intensity vs. *Q*_r_. Three peaks were observed for the cellulose nanofiber and correspond to the (11̅0), (110), and (200) planes of cellulose crystalline planes and fell in the *Q*_r_ ranges of 0.9–1.1, 1.2–1.6, and 1.6–2.0 Å^−1^, respectively. The scattering was integrated with each peak over these ranges to create intensity vs. azimuthal‐angle profiles (*I* vs. χ) for each of the peaks in order to evaluate the orientational order of the fibers in the neat cellulose films and in the cellulose/MXene blends. In addition, the GIWAXS for the neat MXene revealed two peaks at 0.4–0.7 and 1.0–1.8 Å^−1^ corresponding to the (002) and (004) planes of the MXene layers, respectively. The (002) peak of the MXene was observed to shift to a lower Qr value (ca. 0.35 Å^−1^) upon blending with CNF. A region of the 2D scattering image far from any scattering features was used to estimate the background scattering, which was then subtracted from the azimuthal scattering profiles, after which the Hermans orientation parameter (HOP) was calculated.

### 4.3 Mechanical Testing

Tensile testing was performed using an EZ‐SX tensile tester (Shimadzu). Due to the limited area of vacuum‐assisted filtration films, miniature tensile specimens (*n* = 5) were cut to dimensions of 2 mm in width and 25 mm in length, with a gauge length of 15 mm, using a razor blade. The strain rate was set at 50 mm/min, and the sample thickness ranged from 3 to 4 µm. Five specimens were tested per composition (*n* = 5). If a specimen exhibited visible macroscopic defects (edge tear or slip) during mounting or failure outside the gauge section, it was discarded, and the test was repeated to maintain *n* = 5 valid specimens. The maximum stress and the strain at which stress began to decrease were recorded as the tensile strength and elongation at break, respectively. The elastic modulus was calculated using the maximum values of the first derivative of the stress–strain curve.

### 4.4 Microscopy

The high‐resolution scanning electron microscopy (SEM) images of CNF‐MXene films were collected using the 8230 FE‐SEM equipped with a cold field emission (CFE) gun with secondary electron (SE) detection at 3 kV and a working distance of 5–15 mm. The samples were attached to a carbon tape on a vertical SEM stub and coated with a 10 nm layer of Au:Pd using Q150V Plus Automatic Coater.

Atomic force microscopy (AFM) was employed to examine the surface topography of the CNF‐MXene samples on a Bruker Dimension Icon microscope operated in tapping mode [49]. Probes from Mikro‐masch, specifically the HQ:XSC11/Hard/Al BS model, using cantilever C with a nominal radius of <20 nm. The scanning rate was 0.6 Hz, and the image resolution was 512 × 512 pixels. Image processing and analysis were conducted using Nanoscope Analysis or Gwyddion Software. Before scanning, as‐synthesized films were attached to double‐sided scotch tape on a glass slide.

Optical microscopy with the Olympus BX51 system enabled characterization of the visual appearance of the obtained CNF‐MXene films’ surface and the distribution of MXene flakes within the transparent cellulose matrix in bright‐light, unpolarized mode, as well as with a fluorescence 500 nm filter, after Rh 6G dye capture, with exposure times of 50–200 ms. The samples were attached to a double‐sided scotch tape on a glass slide and placed on a motorized stage to enable precise scanning across the area of interest.

### 4.5 Toxicity Assessment

Mouse embryonic fibroblasts (BALB/3T3 clone A31, ATCC CCL‐163) at passage 54 were used to assess biocompatibility. Cells were maintained in DMEM (Sigma–Aldrich) supplemented with 10% fetal bovine serum, 100 U mL^−^^1^ penicillin, and 100 µg mL^−^^1^ streptomycin (Gibco) at 37°C, 5% CO_2_. STR profiling confirmed line authenticity and absence of mycoplasma. Circular CNF‐MXene membranes (Ø 5 mm) were UV‐sterilized for 20 min per side, keeping the films separated to ensure full exposure. Cells expanded in T‐75 flasks were seeded onto the membranes (10,000 cells cm^−^^2^) in 96‐well plates (200 µL medium well^−^^1^). After 24 h, the membranes were transferred to fresh wells to avoid signal from off‐sample cells. Metabolic activity after 72 h was quantified using a resazurin reduction assay: resazurin solution (0.15 mg mL^−^^1^, pH 7.4) was added at 10% of the well volume, incubated for 2 h, and fluorescence was read at 530 nm/590 nm (Multiskan FC). Triplicate samples, a positive control (cells only), and a negative control (medium only) were included. For fluorescence imaging on day 3, samples were fixed and stained as described previously [50], and visualized with a Nikon Eclipse Ti microscope using DAPI and TRITC channels.

### 4.6 Antimicrobial Tests

A *Staphylococcus aureus* suspension (10^6^ CFU mL^−^^1^, 200 µL per well) was introduced into 96‐well plates containing UV‐sterilized CNF‐MXene discs and incubated in Mueller–Hinton broth (MHB) for 18 h at 37°C; wells with inoculum only (growth control) and MHB alone (sterile control) were included. After incubation, the entire plate was irradiated with an 808 nm diode laser (DEN5A, Gigaa Optronics; 2 W, 10 Hz, 5 min). Immediately afterward, 10 µL from each well were spread on Mueller–Hinton agar (MHA) to quantify surviving planktonic cells. For biofilm assessment, each disc was rinsed three times with PBS to remove loosely attached cells, then transferred to 10 mL PBS and sonicated for 1 min (Branson B3500S‐MT) to detach adherent bacteria; 10 µL of the resulting suspension was streak‐plated on MHA and incubated for 24 h at 37°C. Antibacterial efficacy was expressed as percentage reduction in colony‐forming units relative to the MXene‐free control using the formula:

(1) $$Survival,\;\%\;=\frac{E}{C}\times 100\%$$

where *E* (CFU mL^−^^1^) is the viable count from the MXene‐containing samples and *C* (CFU mL^−^^1^) is the count from MXene‐free controls. A lower *E* relative to *C* indicates effective photothermal inactivation.

### 4.7 Dye Adsorption

A Rhodamine 6G dye (Sigma–Aldrich) stock solution was prepared by dissolving 10 mg of dye in 10 mL of deionized water to obtain a 40 µm concentration, which was then serially diluted to generate a calibration set (e.g., 5–20 mg/L). A Beer‐Lambert linear calibration curve enabled conversion of absorbance intensity to concentration for all samples (R^2^ = 0.997). Each studied film sample was cut into four equal‐mass pieces and placed separately in glass vials containing 20 mL of Rh 6G with an initial concentration C_0_ of ∼ 5.5 mg/L, then covered with aluminum foil to prevent photobleaching. Vials were gently agitated to minimize boundary layers without damaging the films and kept steady on the lab bench. At set times *t* (e.g., 0, 20, 40 min, 1 h, 2 h, 7 h, 24 h, 48 h, 72 h), 3 mL aliquots were withdrawn and measured by a Shimadzu UV‐3600 Plus spectrophotometer. Aliquots were returned after reading to preserve the volume of the tested solution. UV–Vis absorbance was collected in plastic cuvettes from 300 to 800 nm for solutions and from 200 to 900 nm for solid films; peak absorbance at ∼ 526 nm was used for quantification.

## Conflicts of Interest

The authors declare no conflicts of interest.

## Supporting information




**Supporting File**: smll74871‐sup‐0001‐SuppMat.pdf.

## Data Availability

The data that supports the findings of this study are available in the supplementary material of this article.

## References

[1] H. Zhao , Z. Yang , and L. Guo , “Nacre‐Inspired Composites With Different Macroscopic Dimensions: Strategies for Improved Mechanical Performance and Applications,” NPG Asia Materials 10 (2018): 1–22, 10.1038/s41427-018-0009-6.

[2] R. Xiong , J. Luan , S. Kang , C. Ye , S. Singamaneni , and V. V. Tsukruk , “Biopolymeric Photonic Structures: Design, Fabrication, and Emerging Applications,” Chemical Society Reviews 49 (2020): 983–1031, 10.1039/C8CS01007B. 31960001

[3] B. Dimitrov , D. Bukharina , V. Poliukhova , et al., “Printed Twisted Thin Films With Near‐Infrared Bandgaps and Tailored Chiroptical Properties,” ACS Applied Optical Materials 2, no. 12 (2024): 2540–2550, 10.1021/acsaom.4c00386. PMC11686506 39744473

[4] B. Dimitrov , V. Poliukhova , C. Joo , et al., “Vivid Structural Coloration in Transparent MXene‐Cellulose Nanocrystals Composite Films,” Advanced Functional Materials 35 (2025): 2420853, 10.1002/adfm.202420853.

[5] K. Adstedt , F. Stojcevski , B. Newman , et al., “Carbon Fiber Surface Functional Landscapes: Nanoscale Topography and Property Distribution,” ACS Applied Materials & Interfaces 14, no. 3 (2022): 4699–4713, 10.1021/acsami.1c20686. 35015495

[6] M.‐C. Hsieh , H. Koga , K. Suganuma , and M. Nogi , “Hazy Transparent Cellulose Nanopaper,” Scientific Reports 7 (2017): 41590, 10.1038/srep41590. PMC5269665 28128326

[7] Z. Fang , G. Hou , C. Chen , and L. Hu , “Nanocellulose‐Based Films and Their Emerging Applications,” Current Opinion in Solid State and Materials Science 23 (2019): 100764, 10.1016/j.cossms.2019.07.003.

[8] R. Das , T. Lindström , P. R. Sharma , K. Chi , and B. S. Hsiao , “Nanocellulose for Sustainable Water Purification,” Chemical Reviews 122 (2022): 8936–9031, 10.1021/acs.chemrev.1c00683. 35330990

[9] M. A. Hubbe , A. Ferrer , P. Tyagi , et al., “Nanocellulose in Thin Films, Coatings, and Plies for Packaging Applications: A Review,” BioResources 12 (2017): 2143–2233, 10.15376/biores.12.1.Hubbe.

[10] S. Kalia , A. Dufresne , B. M. Cherian , et al., “Cellulose‐Based Bio‐ and Nanocomposites: A Review,” International Journal of Polymer Science 2011 (2011): 1–35, 10.1155/2011/837875.

[11] M. Naguib , M. Kurtoglu , V. Presser , et al., “Two‐Dimensional Nanocrystals Produced by Exfoliation of Ti_3_AlC_2_ ,” Advanced Materials 23 (2011): 4248–4253, 10.1002/adma.201102306. 21861270

[12] Y. Gogotsi , “The Future of MXenes,” Chemistry of Materials 35 (2023): 8767–8770, 10.1021/acs.chemmater.3c02491.

[13] A. Lipatov , H. Lu , M. Alhabeb , et al., “Elastic Properties of 2D Ti_3_C_2_T_x_ MXene Monolayers and Bilayers,” Science Advances 4 (2018): aat0491, 10.1126/sciadv.aat0491. PMC6003751 29922719

[14] S. Luo , S. Patole , S. Anwer , et al., “Tensile Behaviors of Ti_3_C_2_T_x_ (MXene) Films,” Nanotechnology 31 (2020): 395704, 10.1088/1361-6528/ab94dd. 32434169

[15] J. Zhang , N. Kong , S. Uzun , et al., “Scalable Manufacturing of Free‐Standing, Strong Ti_3_C_2_T_x_ MXene Films With Outstanding Conductivity,” Advanced Materials 32 (2020): 2001093, 10.1002/adma.202001093. 32309891

[16] J. FitzPatrick and Y. Gogotsi , “MXene Polymer Composites: A Review,” MRS Bulletin 50 (2025): 1351–1363, 10.1557/s43577-025-00984-x.

[17] T. Yuan , Z. Zhang , Q. Liu , X.‐T. Liu , Y.‐N. Miao , and C. Yao , “MXene (Ti_3_C_2_T_x_)/Cellulose Nanofiber/Polyaniline Film as a Highly Conductive and Flexible Electrode Material for Supercapacitors,” Carbohydrate Polymers 304 (2023): 120519, 10.1016/j.carbpol.2022.120519. 36641165

[18] Z. Zhan , Q. Song , Z. Zhou , and C. Lu , “Ultrastrong and Conductive MXene/Cellulose Nanofiber Films Enhanced by Hierarchical Nano‐Architecture and Interfacial Interaction for Flexible Electromagnetic Interference Shielding,” Journal of Materials Chemistry C 7 (2019): 9820–9829, 10.1039/C9TC03309B.

[19] B. Zhou , Z. Zhang , Y. Li , et al., “Flexible, Robust, and Multifunctional Electromagnetic Interference Shielding Film with Alternating Cellulose Nanofiber and MXene Layers,” ACS Applied Materials & Interfaces 12 (2020): 4895–4905, 10.1021/acsami.9b19768. 31898463

[20] W.‐T. Cao , F.‐F. Chen , Y.‐J. Zhu , et al., “Binary Strengthening and Toughening of MXene/Cellulose Nanofiber Composite Paper With Nacre‐Inspired Structure and Superior Electromagnetic Interference Shielding Properties,” ACS Nano 12 (2018): 4583–4593, 10.1021/acsnano.8b00997. 29709183

[21] S. Zeng , Y. Ye , P. Zhou , et al., “Programmable and Reconfigurable Humidity‐Driven Actuators Made With MXene (Ti_3_C_2_T_x_)‐Cellulose Nanofiber Composites for Biomimetic Applications,” Nano Research 17 (2024): 6619–6629, 10.1007/s12274-024-6542-4.

[22] D. Liu , Y. Gao , Y. Song , et al., “Highly Sensitive Multifunctional Electronic Skin Based on Nanocellulose/MXene Composite Films With Good Electromagnetic Shielding Biocompatible Antibacterial Properties,” Biomacromolecules 23 (2022): 182–195, 10.1021/acs.biomac.1c01203. 34889593

[23] S. Hao , H. Han , Z. Yang , et al., “Recent Advancements on Photothermal Conversion and Antibacterial Applications Over MXenes‐Based Materials,” Nano‐Micro Letters 14 (2022): 178, 10.1007/s40820-022-00901-w. PMC9402885 36001173

[24] K. Li , C. M. Clarkson , L. Wang , et al., “Alignment of Cellulose Nanofibers: Harnessing Nanoscale Properties to Macroscale Benefits,” ACS Nano 15 (2021): 3646–3673, 10.1021/acsnano.0c07613. 33599500

[25] W. Tian , A. VahidMohammadi , M. S. Reid , et al., “Multifunctional Nanocomposites With High Strength and Capacitance Using 2D MXene and 1D Nanocellulose,” Advanced Materials 31 (2019): 1902977, 10.1002/adma.201902977. 31408235

[26] X. Ma , A. Wang , X. Zhang , et al., “Photo‐Crosslinking Injectable Photothermal Antibacterial Hydrogel Based on Quaternary Ammonium Grafted Chitosan and Hyaluronic Acid for Infected Wound Healing,” Materials Today Bio 29 (2024): 101265, 10.1016/j.mtbio.2024.101265. PMC11866169 40018434

[27] V. Poliukhova , B. Dimitrov , J. Brackenridge , et al., “Multispectral Photonic Structural Colors via Enhanced Interfacial Interference of Ultrathin Cellulose Nanofiber/MXene Films,” Advanced Science 12: 2500953, 10.1002/advs.202500953. PMC12199417 40192439

[28] K. M. O. Håkansson , A. B. Fall , F. Lundell , et al., “Hydrodynamic Alignment and Assembly of Nanofibrils Resulting in Strong Cellulose Filaments,” Nature Communications 5 (2014): 4018, 10.1038/ncomms5018. PMC4059937 24887005

[29] C. Kamal , S. Gravelle , and L. Botto , “Hydrodynamic Slip Can Align Thin Nanoplatelets in Shear Flow,” Nature Communications 11 (2020): 2425, 10.1038/s41467-020-15939-w. PMC7229003 32415194

[30] X. Han , P. Chen , L. Li , Y. Nishiyama , and X. Yang , “Planar and Uniplanar Orientation in Nanocellulose Films: Interpretation of 2D Diffraction Patterns Step‐by‐Step,” Cellulose 30 (2023): 8151–8159, 10.1007/s10570-023-05411-5.

[31] M. van Drongelen , D. Cavallo , L. Balzano , et al., “Structure Development of Low‐Density Polyethylenes During Film Blowing: A Real‐Time Wide‐Angle X‐ray Diffraction Study,” Macromolecular Materials and Engineering 299 (2014): 1494–1512, 10.1002/mame.201400161.

[32] W. Yang , V. R. Sherman , B. Gludovatz , et al., “On the Tear Resistance of Skin,” Nature Communications 6 (2015): 6649, 10.1038/ncomms7649. PMC4389263 25812485

[33] Y. Liu , W. Zou , N. Zhao , and J. Xu , “Electrically Insulating PBO/MXene Film With Superior Thermal Conductivity, Mechanical Properties, Thermal Stability, and Flame Retardancy,” Nature Communications 14 (2023): 5342, 10.1038/s41467-023-40707-x. PMC10475028 37660170

[34] X. Zhang , S. Kang , K. Adstedt , et al., “Uniformly Aligned Flexible Magnetic Films From Bacterial Nanocelluloses for Fast Actuating Optical Materials,” Nature Communications 13 (2022): 5804, 10.1038/s41467-022-33615-z. PMC9530119 36192544

[35] J. Tang , R. Zhu , Y.‐H. Pai , Y. Zhao , C. Xu , and Z. Liang , “Thermoelectric Modulation of Neat Ti_3_C_2_T_x_ MXenes by Finely Regulating the Stacking of Nanosheets,” Nano‐Micro Letters 17 (2025): 93, 10.1007/s40820-024-01594-z. PMC11671675 39724483

[36] W. Chen , D. Zhang , K. Yang , M. Luo , P. Yang , and X. Zhou , “Mxene (Ti_3_C_2_T_x_)/Cellulose Nanofiber/Porous Carbon Film as Free‐Standing Electrode for Ultrathin and Flexible Supercapacitors,” Chemical Engineering Journal 413 (2021): 127524, 10.1016/j.cej.2020.127524.

[37] F. Zhang , P. Ren , Z. Guo , et al., “Asymmetric Multilayered MXene‐AgNWs/Cellulose Nanofiber Composite Films With Antibacterial Properties for High‐Efficiency Electromagnetic Interference Shielding,” Journal of Materials Science and Technology 129 (2022): 181–189.

[38] K. Rasool , M. Helal , A. Ali , C. E. Ren , Y. Gogotsi , and K. A. Mahmoud , “Antibacterial Activity of Ti_3_C_2_T_x_ MXene,” ACS Nano 10 (2016): 3674–3684, 10.1021/acsnano.6b00181. 26909865

[39] K. Rasool , K. A. Mahmoud , D. J. Johnson , M. Helal , G. R. Berdiyorov , and Y. Gogotsi , “Efficient Antibacterial Membrane based on Two‐Dimensional Ti_3_C_2_T_x_ (MXene) Nanosheets,” Scientific Reports 7 (2017): 1598, 10.1038/s41598-017-01744-2. PMC5431673 28487521

[40] R. Li , L. Zhang , L. Shi , and P. Wang , “MXene Ti_3_C_2_: An Effective 2D Light‐to‐Heat Conversion Material,” ACS Nano 11 (2017): 3752–3759, 10.1021/acsnano.6b08415. 28339184

[41] K. P. Marquez , K. M. D. Sisican , R. P. Ibabao , et al., “Understanding the Chemical Degradation of Ti_3_C_2_T_x_ MXene Dispersions: A Chronological Analysis,” Small Science 4 (2024): 2400150, 10.1002/smsc.202400150. PMC11935063 40212246

[42] J. Yoon , S. Kim , K. H. Park , et al., “Biocompatible and Oxidation‐Resistant Ti_3_C_2_T_x_ MXene With Halogen‐Free Surface Terminations,” Small Methods 7 (2023): 2201579, 10.1002/smtd.202201579. 36929585

[43] J. Wu , Y. Yu , and G. Su , “Safety Assessment of 2D MXenes: In Vitro and In Vivo,” Nanomaterials 12 (2022): 828, 10.3390/nano12050828. PMC8912767 35269317

[44] Y. Cai , H. Gao , Y. Qu , et al., “Photothermal/Photodynamic Synergistic Antibacterial Nanocellulose Film Modified With Antioxidant MXene‐PANI Nanosheets,” International Journal of Biological Macromolecules 300 (2025): 140283, 10.1016/j.ijbiomac.2025.140283. 39863226

[45] E. Jiao , K. Wu , Y. Liu , et al., “Robust Bioinspired MXene‐Based Flexible Films With Excellent Thermal Conductivity and Photothermal Properties,” Composites Part A: Applied Science and Manufacturing 143 (2021): 106290, 10.1016/j.compositesa.2021.106290.

[46] F. Wu , H. Zheng , W. Wang , et al., “Rapid Eradication of Antibiotic‐Resistant Bacteria and Biofilms by MXene and Near‐Infrared Light Through Photothermal Ablation,” Science China Materials 64 (2021): 748–758, 10.1007/s40843-020-1451-7.

[47] T. Habib , X. Zhao , S. A. Shah , et al., “Oxidation Stability of Ti_3_C_2_T_x_ MXene Nanosheets In Solvents and Composite Films,” npj 2D Materials and Applications 3 (2019): 8, 10.1038/s41699-019-0089-3.

[48] W. Zhao , H. Chi , S. Zhang , X. Zhang , and T. Li , “One‐Pot Synthesis of Cellulose/MXene/PVA Foam for Efficient Methylene Blue Removal,” Molecules 27 (2022): 4243, 10.3390/molecules27134243. PMC9268378 35807488

[49] M. E. McConney , S. Singamaneni , and V. V. Tsukruk , “Probing Soft Matter With the Atomic Force Microscopies: Imaging and Force Spectroscopy,” Polymer Reviews 50 (2010): 235–286, 10.1080/15583724.2010.493255.

[50] K. Diedkova , A. D. Pogrebnjak , S. Kyrylenko , et al., “Polycaprolactone–MXene Nanofibrous Scaffolds for Tissue Engineering,” ACS Applied Materials & Interfaces 15 (2023): 14033–14047, 10.1021/acsami.2c22780. 36892008

