# Supplementary material for: Multifunctional Cellulose Nanofiber Films With Embedded Highly Oriented MXene Flakes
Source: Small. 2026 Aug 4;22(54):e74871. doi: 10.1002/smll.74871 (PMC13614276; doi:10.1002/smll.74871)
Supplement: Supplementary file 1 [file SMLL-22-e74871-s001.pdf]

Supporting Information

Multifunctional Composite Cellulose Nanofiber Films with Embedded Highly Oriented MXene Flakes

Valeriia Poliukhova[1], Jacob Crossno[2,3], Kateryna Diedkova[4,5], Viktoriia Korniienko[4,5], Jisoo Jeon[1], Sankarshanaram S Vempati[1], Jaejun Lee[1], Kirt A. Page[2,3], Hilmar Koerner[2], Maksym Pogorielov[4,5], Yury Gogotsi[6], Vladimir V. Tsukruk[1] *

1 School of Materials Science and Engineering, Georgia Institute of Technology, Atlanta, Georgia 30332, United States

2 Materials and Manufacturing Directorate, Air Force Research Laboratory, WPAFB, Dayton, OH 45433, United States

3 AV Inc., Dayton, OH 45432, United States

4 Biomedical Research Center, Medical Institute, Sumy State University, 31 Sanatornaya Str., 40018 Sumy, Ukraine

5 Institute of Atomic Physics and Spectroscopy, University of Latvia, 3 Jelgavas Str., LV-1004 Riga, Latvia

6 A. J. Drexel Nanomaterials Institute and Department of Materials Science and Engineering, Drexel University, Philadelphia, Pennsylvania 19104, United States

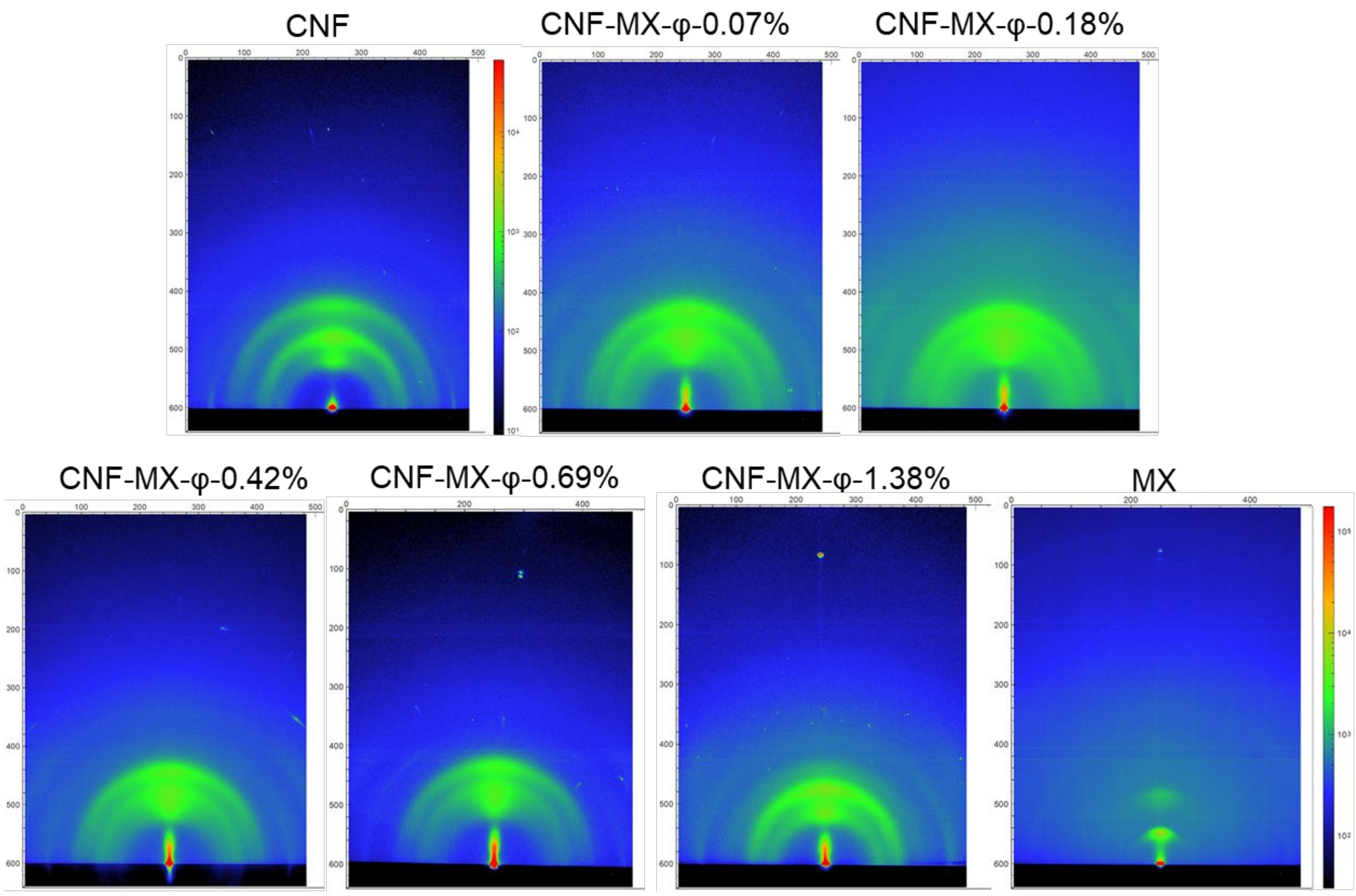

Figure S1. In-plane GIWAXS 2D scattering images for all samples reported in the manuscript.

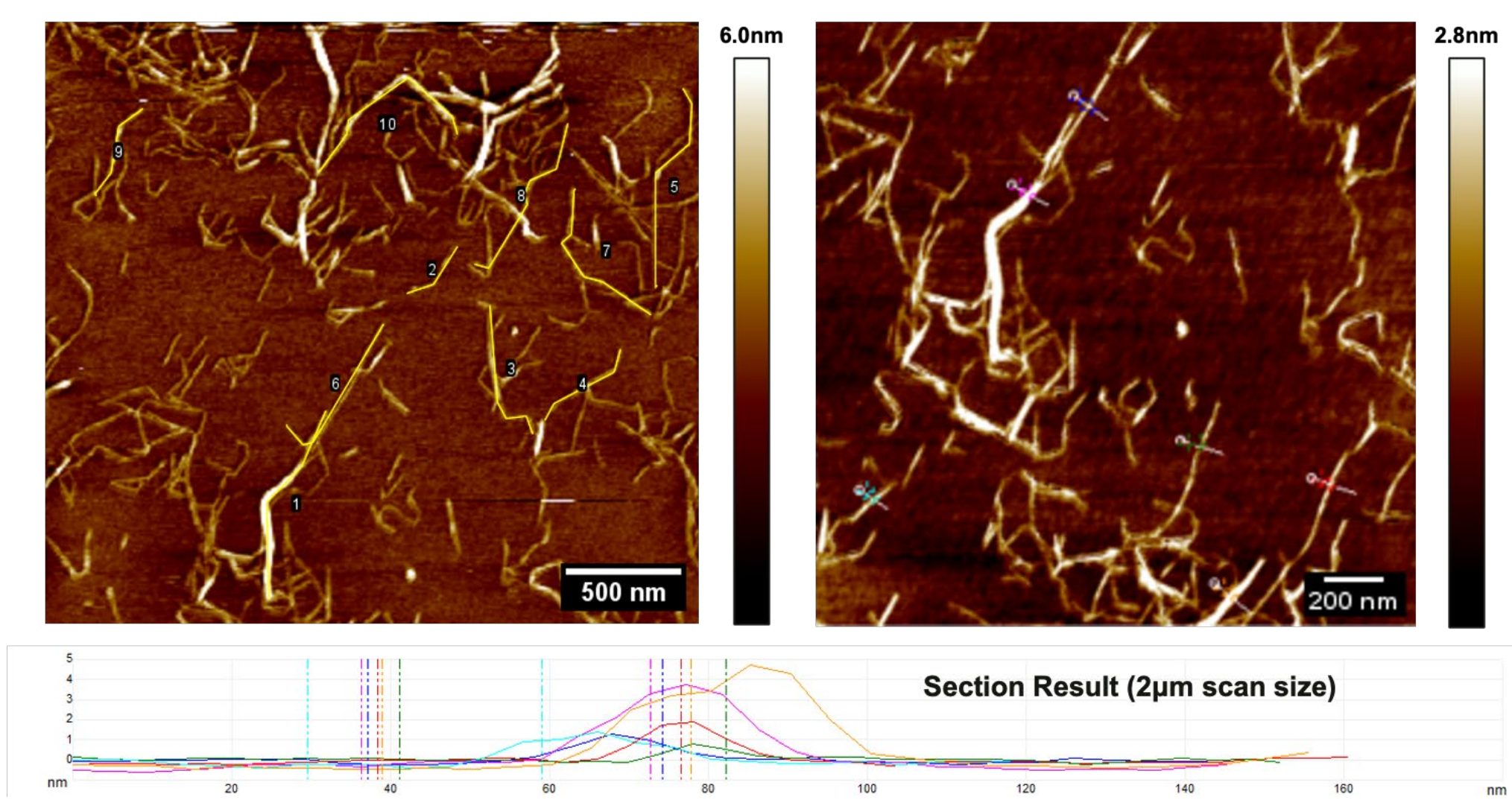

Figure S2. AFM topography images of CNF used to prepare CNF-MXene films with average diameter of 1.8 nm and an average length of 700 nm, scan size is 5 and 2 μm.

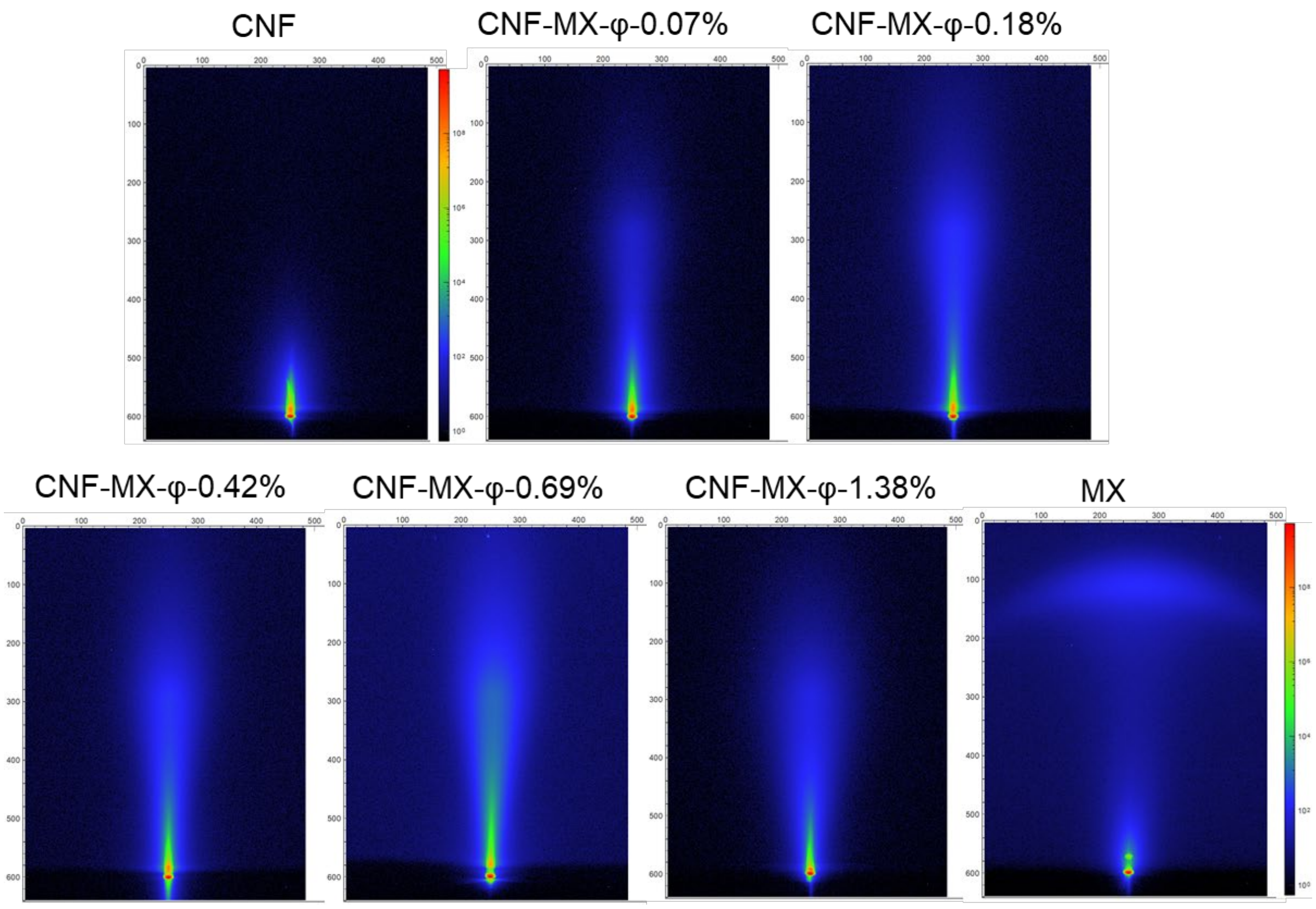

Figure S3. In-plane GIMAXS 2D scattering images for CNF, CNF-MXene and MXene films.

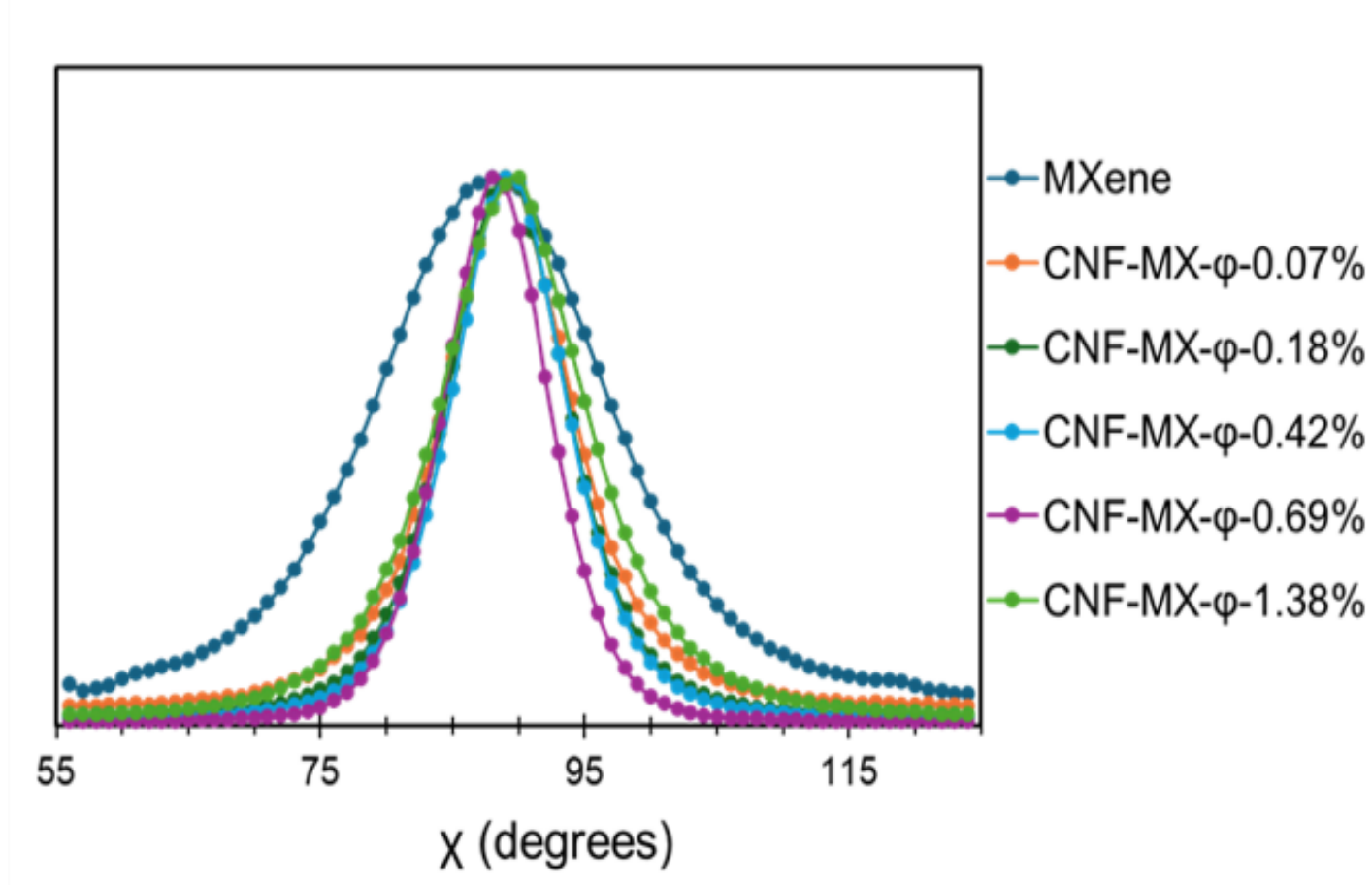

Figure S4. A comparison of the MXene (002) azimuthal peak widths from the GIMAXS for MXene from selected CNF-MX-φ samples showing azimuthal intensity profiles (I vs χ).

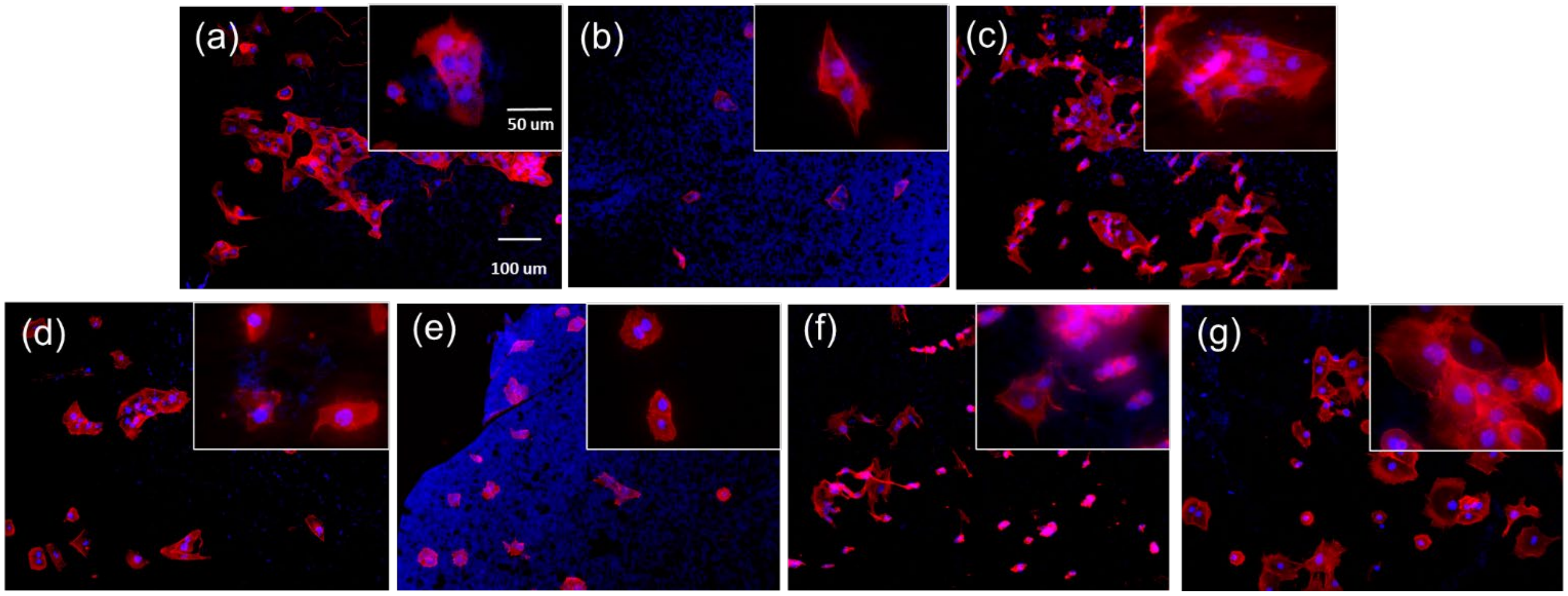

Figure S5. Fluorescent images of nuclei (blue) and cytoskeleton staining (red) on the 3rd day of cultures on the *CNF-MX-φ-0.07%-1.38%* films (a-g) with higher magnification images in insets, respectively

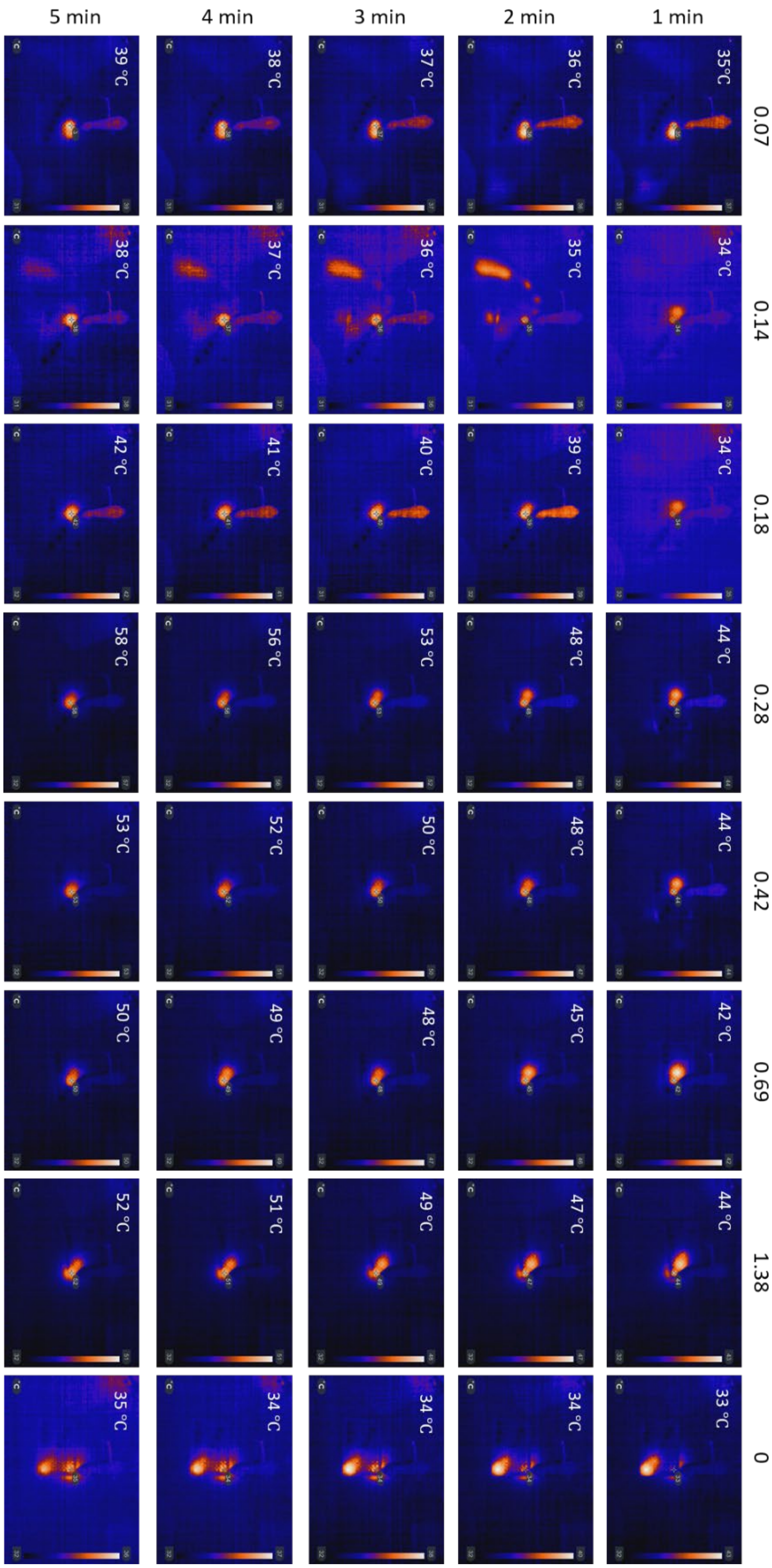

Figure S6. Recorded images of the temperature at 1-5 minutes of 808 nm at 2 watts/10 Hz laser exposure on the *CNF-MX-φ-0.07%-1.38%* films and CNF.

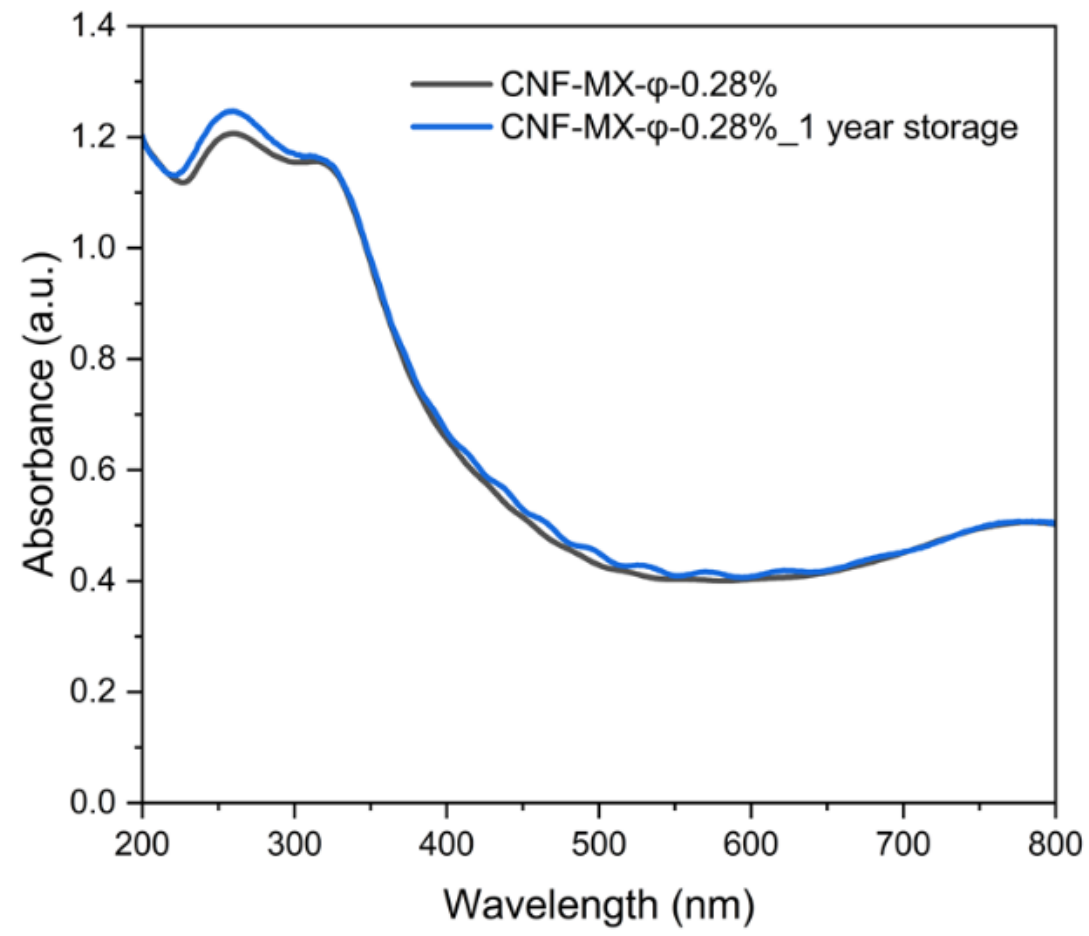

Figure S7. UV-Vis spectra of the *CNF-MX-φ-0.28%* film after the synthesis and fabrication process (black line) and after one year of storage (blue line).

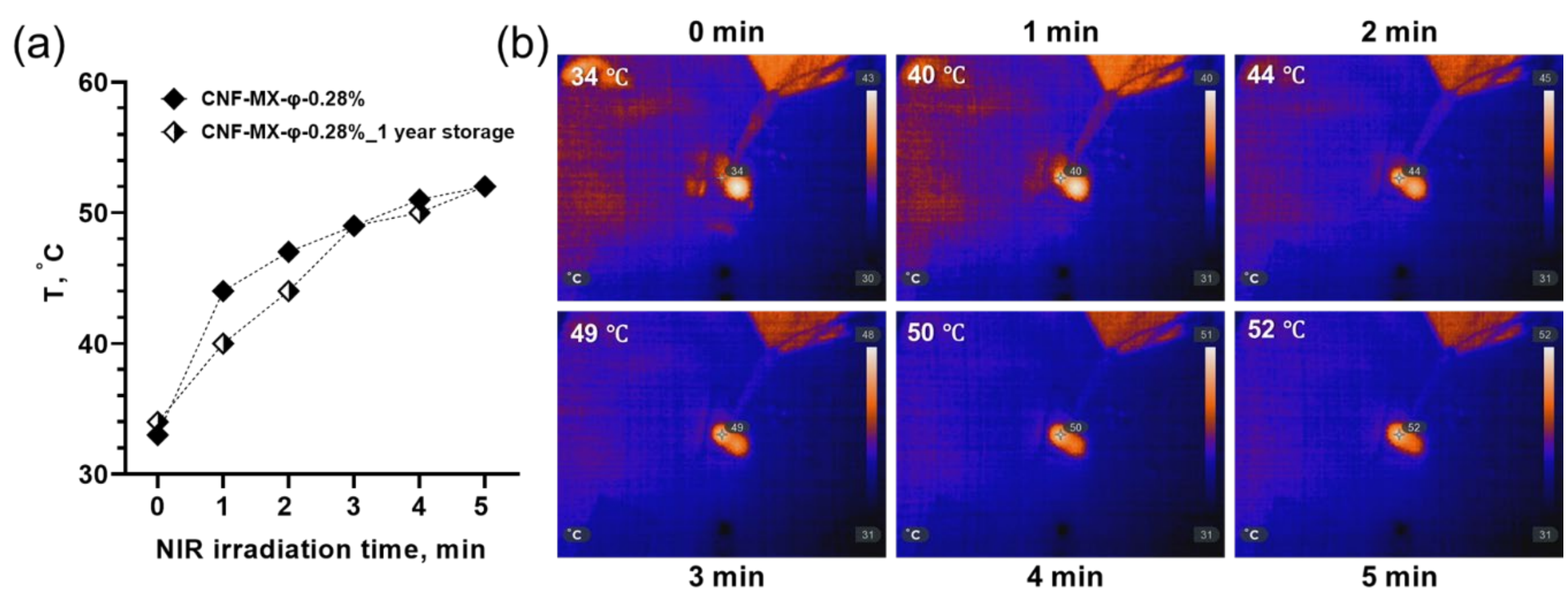

Figure S8. Recorded images of the temperature rise at 1-5 minutes of 808 nm at 2 watts/ 10 Hz laser exposure on the *CNF-MX-φ-0.28%* film before and after 1 year of ambient dark conditions storage, performed under the same experimental conditions.

Table S1. Calculated Herman's orientation parameter (HOP) from GIWAXS for (110) and (200) CNF reflection peaks.

**Sample Name**	**(110) Peak**		**(200) Peak**	
	$\mathbf{Q_{110}}$	$\mathbf{D_{110}}$	$\mathbf{Q_{200}}$	$\mathbf{D_{200}}$
	1.35 $Å^{-1}$	4.65 Å	1.87 $Å^{-1}$	3.36 Å
	**HOP**		**HOP**	
Neat CNF	0.47		0.40	
CNF-MX-φ-0.07%	0.49		0.46	
CNF-MX-φ-0.18%	0.50		0.49	
CNF-MX-φ-0.42%	0.48		0.49	
CNF-MX-φ-0.69%	0.53		0.48	
CNF-MX-φ-1.38%	0.38		0.40	

Table S2. Calculated Herman's orientation parameter (HOP) from GIMAXS/GIWAXS for (002) MXene reflection peak in MXene and from GIMAXS for CNF-MXene films.

**Sample Name**	**(002) Peak**	
	$\mathbf{Q_{002}}$	$\mathbf{D_{002}}$
	0.60 $Å^{-1}$	10.47 Å
	**HOP**	
Neat MXene	0.70 (WAXS)/0.85 (MAXS)	
**CNF/MXene Blends**	**(002) Peak**	
	$\mathbf{Q_{002}}$	$\mathbf{D_{002}}$
	0.35 $Å^{-1}$	17.95 Å
CNF-MX-φ-0.07%	0.88	
CNF-MX-φ-0.18%	0.92	
CNF-MX-φ-0.42%	0.93	
CNF-MX-φ-0.69%	0.94	
CNF-MX-φ-1.38%	0.90	

Table S3. Mean values of the mechanical properties of CNF-MXene composite films.

Sample	Modulus (GPa)	Tensile strength (MPa)	Elongation at break (%)	Toughness (MJ/m^3)
CNF	3 ± 1	62.6 ± 25.3	4.3 ± 1.4	1.4 ± 1.0
CNF-MX-φ-0.07%	8.5 ± 1.2	222.4 ± 28.8	8.7 ± 1.9	13.7 ± 4.5
CNF-MX-φ-0.14%	7.5 ± 2.8	195.3 ± 20.2	8.1 ± 3.6	11.5 ± 6.7
CNF-MX-φ-0.18%	9.3 ± 1.5	221.6 ± 11.5	7.1 ± 1	10.5 ± 1.9
CNF-MX-φ-0.28%	8.1 ± 0.8	230.5 ± 16.5	5.3 ± 0.7	8.3 ± 1.4
CNF-MX-φ-0.42%	7.8 ± 1.5	193 ± 40.6	4.2 ± 1.8	5.6 ± 3.4
CNF-MX-φ-0.69%	10 ± 3.9	253.1 ± 41	4.9 ± 0.6	8.1 ± 2.3
CNF-MX-φ-1.38%	7.98 ± 1.97	240.7 ± 16.6	5.2 ± 1.2	7.4 ± 1.9
MXene	3.4 ± 1	15.7 ± 8.4	1 ± 0.2	0.08 ± 0.06
